# Human activities favour prolific life histories in both traded and introduced vertebrates

**DOI:** 10.1038/s41467-022-35765-6

**Published:** 2023-01-17

**Authors:** Sally E. Street, Jorge S. Gutiérrez, William L. Allen, Isabella Capellini

**Affiliations:** 1 Department of Anthropology, Durham University, Durham, DH1 3LE UK; 2 Department of Anatomy, Cell Biology and Zoology, University of Extremadura, Badajoz, 06006 Spain; 3 Department of Biosciences, Swansea University, Swansea, SA2 8PP UK; 4 School of Biological Sciences, Queens University Belfast, Belfast, BT9 5DL UK

**Keywords:** Invasive species, Conservation biology, Evolutionary ecology, Macroecology

## Abstract

Species’ life histories determine population demographics and thus the probability that introduced populations establish and spread. Life histories also influence which species are most likely to be introduced, but how such ‘introduction biases’ arise remains unclear. Here, we investigate how life histories affect the probability of trade and introduction in phylogenetic comparative analyses across three vertebrate classes: mammals, reptiles and amphibians. We find that traded species have relatively high reproductive rates and long reproductive lifespans. Within traded species, introduced species have a more extreme version of this same life history profile. Species in the pet trade also have long reproductive lifespans but lack ‘fast’ traits, likely reflecting demand for rare species which tend to have slow life histories. We identify multiple species not yet traded or introduced but with life histories indicative of high risk of future trade, introduction and potentially invasion. Our findings suggest that species with high invasion potential are favoured in the wildlife trade and therefore that trade regulation is crucial for preventing future invasions.

## Abstract (web summary)

Predicting which species will become invasive is vital because the harm they cause cannot always be mitigated once populations establish. Street et al. show that traded and introduced species have distinctive life histories with high invasion potential, helping to identify future invasion risks.

## Introduction

Alien invasive species cause substantial biodiversity loss, disruption to ecosystem services and staggering economic costs worldwide^1–3^. Despite increased awareness of the harm caused by alien invasive species, the rate of new introductions continues to accelerate in many taxa^4^. Understanding why some species are more likely than others to become invaders is essential for effective management because controlling alien populations once established and spread can be prohibitively difficult and expensive^5,6^. Most research to date has focused on the drivers of success at the final two stages of the invasion process, establishment and spread, at the expense of the first two, transportation and introduction (i.e. the release of non-native species into the wild by humans either deliberately or accidentally)^6,7^. However, the selection of species for introduction is highly biased: introduced species systematically differ from non-introduced species in terms of taxonomy, geographic origin and biological characteristics (e.g. Refs. ^8–12^). These ‘introduction biases’ determine which species have the opportunity to become future invaders^6,9,12^ and may have a major influence on outcomes at later invasion stages, especially if they favour the introduction of species with characteristics that predispose them towards successful establishment and spread. Therefore, identifying at what stage of the invasion pathway introduction biases arise is essential for preventing future invasions.

The accelerating rate of alien introductions and their associated costs in recent decades is largely the result of increasing international trade^3,4,13^. The live wildlife trade, particularly the burgeoning international pet trade, is increasingly the predominant introduction pathway for vertebrates, especially reptiles and amphibians^14–17^, despite increasing wildlife trade restrictions^18,19^. For example, slider turtles (*Trachemys scripta*) have been released in over 70 countries worldwide, primarily via the pet trade^20^, while released pet Burmese pythons (*Python bivittatus*) are the likely cause of dramatic population reductions in several native mammal species in the Florida Everglades^21^. Given the importance of trade as a pathway to introduction, introduction biases may be explained by human preferences for which species to trade and transport, rather than for which species to release. For example, taxonomic biases among introduced birds and fishes likely reflect the popularity of certain groups for recreational hunting^11,12^, and large body size in introduced vertebrates may be explained by the appeal of larger species for a variety of common human uses, such as food, pets or biocontrol^8,9,11,12,15,22^. However, we do not yet know at what stage introduction biases arise since very few studies distinguish biases associated with transportation from those associated with introduction^7^. The distinction between these two stages is, however, crucial as it represents the point at which species are no longer contained in captivity and have the opportunity to survive and reproduce in the wild^7^. If biases differ between the two stages, lumping them together in analyses of invasion success risks inaccurate predictions for future invasions and misinformed management strategies.

Species’ life histories—evolved strategies of development and reproduction over an organism’s lifetime—play a central role in biological invasions since they influence population growth rates and thus the ability of small founder populations to survive and expand in novel environments^23,24^. ‘Fast’ life history traits, such as rapid maturation and high reproductive rates, increase the probability of establishment and spread among introduced mammals, reptiles, amphibians and some plant taxa^25–28^ while ‘bet-hedging’ strategies, which involve adjusting investment in reproduction over the lifespan in response to changing environmental conditions—promote establishment in birds^29^. Life history biases, however, begin earlier in the invasion pathway: introduced alien mammals, reptiles and amphibians have an unusual life history profile that combines some ‘fast’ life history traits (large and/or frequent broods) with a long reproductive (i.e. post-maturational) lifespan^25,26^. Species with this life history strategy are highly prolific, producing very large numbers of offspring across their lifetimes. These introduction biases may be explained by human preferences for highly fecund species in live trade, which are likely most lucrative in industries involving large-scale captive breeding such as the pet, food and fur/skin trades^14,30^. Long-lived, highly fecund species may also be at a higher risk of introduction either because longer-term care commitments increase the risk of deliberate release by private owners or because high fecundity and longer lifespans result in more frequent opportunities for accidental escapes^14,15^. Human activities, therefore, may result in the trade and introduction of species with highly prolific life histories that predispose them towards successful establishment and spread^14^. In support of this concern, a recent analysis demonstrates that invasive species are substantially over-represented among vertebrates in the pet trade^17^. However, since previous studies have rarely separated the transportation and introduction stages, we currently do not know whether life history biases occur at transportation, introduction or both stages, and if the latter, whether biases are consistent across the two stages.

Here, we investigate the role of life history traits in the transportation and introduction of alien mammals, reptiles and amphibians in large-scale phylogenetic comparative analyses. Our analyses allow us to identify at which stage life history biases emerge and whether they are consistent across stages. If introduction biases are fully explained by human preferences for species with life histories most lucrative for live trade, we expect that highly prolific life histories (fast traits and long reproductive lifespans) are associated with higher probability and frequency of trade, but not with introduction among traded species. Conversely, if this life history profile increases the risk of introduction but plays no role in species’ appeal for live trade, we should find that it is related only to introduction and not to trade. Alternatively, if the same, highly prolific life history strategy promotes both transportation and introduction, we should find that it predicts both trade and introduction among traded species. We test predictions using data on the live international wildlife trade from the United States Fish and Wildlife Service (USFWS) Law Enforcement Management Information System (LEMIS) database^31^, and separate data on the pet trade specifically from the International Union for Conservation of Nature (IUCN) Red List^32^. We combine these data with our prior, global-scale compilations of introductions and life history traits in mammals, reptiles and amphibians^25,26^. We also obtain species occurrence records^33^, geographic ranges^32^ and population density data^34^ to account for a potentially confounding effect of recording biases: longer-lived, larger-bodied species may be more likely to be recorded in introduction databases as they are more likely to be detected by human observers than shorter-lived, smaller-bodied species. We analyse data using Bayesian generalized linear mixed models (GLMMs), correcting for statistical non-independence due to shared evolutionary history among related species by fitting phylogenetic random effects^35–38^. Further, we use model predictions to investigate to what extent life history traits distinguish traded from non-traded species and (among traded species) introduced from non-introduced species, and thus may be helpful for identifying species at a high risk of trade or introduction in future.

## Results

### Live wildlife trade

**Fig. 1:**
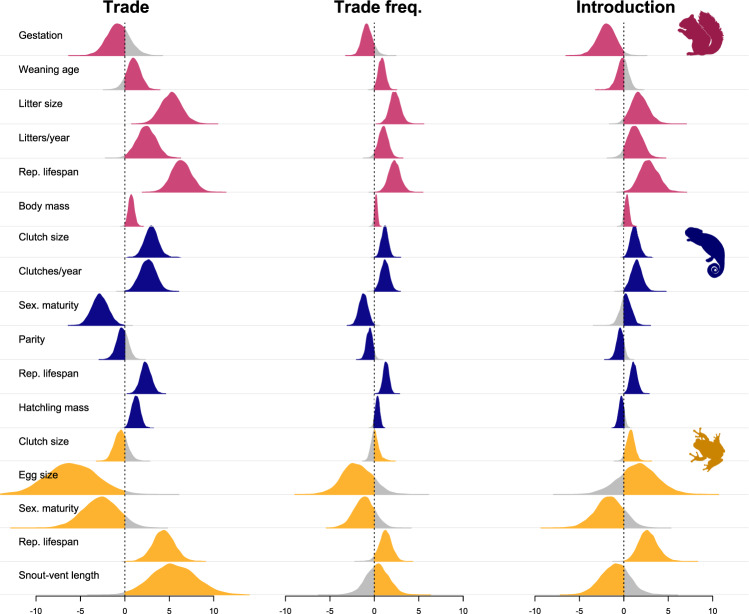
Effects of life history traits on trade, trade frequency and introduction. Posterior distributions of fixed effect estimates from models based on US live wildlife trade data, for effects of life history traits on trade probability (traded vs. non-traded species) and trade frequency (N shipments per species) from hurdle models and introduction (introduced vs. non-introduced species, within traded species only) from probit models across mammals (pink), reptiles (blue) and amphibians (gold). Silhouettes were obtained from phylopic.org under Public Domain licences.

Sample sizes for all analyses are summarised in Table 1. Consistently across mammals, reptiles and amphibians, the probability and/or frequency of live wildlife trade by the US increases with reproductive lifespan and one or more ‘fast’ life history traits (Fig. 1, Tables 2–4). Predictors of the probability of trade are largely consistent between hurdle (Tables 2–4) and probit (Supplementary Tables 2–4) models although effects tended to be slightly stronger in hurdle models. Fast life history traits associated with increased probability and/or frequency of live trade include larger litters in mammals, larger clutches, more frequent clutches and earlier sexual maturity in reptiles, and smaller eggs in amphibians (Fig. 1, Tables 2–4, Supplementary Tables 2–4). Larger body size also increases the probability (but not the frequency) of trade in mammals and amphibians (Fig. 1, Tables 2–4, Supplementary Tables 2–4). Larger hatchling mass increases the probability (with a weaker effect on frequency) of trade in reptiles, which also likely reflects effects of larger adult body size given the high collinearity of adult and hatchling mass in reptiles.

**Table 1:** Sample sizes for analyses

Trade type	Class	Total N species	N traded	N introduced
Live wildlife trade	Mammals	518	312	134
	Reptiles	408	285	141
	Amphibians	132	75	45
Pet trade	Mammals	518	67	22
	Reptiles	408	183	89
	Amphibians	132	48	32

Sample sizes for analyses of the role of life history traits in trade and introduction, separated by trade type taxonomic class. The column ‘Total N species’ refers to species for which we have complete life history data, forming the sample sizes for analyses of life history correlates of trade. The column ‘N traded’ indicates the number of traded species, forming the total sample sizes for analyses of life history correlates of introduction within traded species, while the final column ‘N introduced’ contains the number of introduced species among those traded.

**Table 2:** Life history predictors of US live wildlife trade probability and frequency in mammals from hurdle models

		Posterior mean	l-95% CI	u-95% CI	% crossing 0	VIF
**Binary**	**Gestation period**	−0.74	−3.21	1.50	26.12	4.34
	**Weaning age**	0.97	−0.43	2.61	10.02	2.82
	**Litter size**	5.27	3.02	7.51	0.00	2.78
	**Litters per year**	2.43	0.38	4.42	0.80	2.62
	**Reproductive lifespan**	6.38	4.19	8.49	0.00	3.28
	**Body mass**	0.72	0.14	1.37	0.84	3.29
**Frequency**	**Gestation period**	−0.76	−1.89	0.46	10.30	4.34
	**Weaning age**	0.86	0.11	1.60	1.18	2.82
	**Litter size**	2.37	1.18	3.48	0.00	2.78
	**Litters per year**	1.02	0.01	2.07	2.64	2.62
	**Reproductive lifespan**	2.28	1.00	3.46	0.02	3.28
	**Body mass**	0.17	−0.15	0.47	12.30	3.29

Full parameters for hurdle model predicting US live wildlife trade probability and frequency in mammals, after iteratively removing variables with the highest VIFs until none were >5 (neonatal body mass and age at first birth, *N* = 518, *H*^2^ for binary component = 0.50 [0.25, 0.71], *H*^2^ for Poisson component = 0.16 [0.00, 0.53]). Post. mean = mean β coefficient from posterior distributions, l-95% CI and u-95% CI = lower and upper 95% credible intervals from posterior distributions respectively, % crossing zero = percentage of estimates in the posterior distribution that overlap with zero in the direction opposite to the majority of the distribution, *VIF* variance inflation factors. For ease of interpretation, effects from the binary component of the model are reversed in sign (so that they represent effects on the probability that the outcome variable is 1 or more, rather than 0).

**Table 3:** Life history predictors of US live wildlife trade probability and frequency in reptiles from hurdle models

		Posterior mean	l-95% CI	u-95% CI	% crossing 0	VIF
**Binary**	**Clutch size**	2.99	1.53	4.39	0.00	1.57
	**Clutches per year**	2.59	0.84	4.17	0.10	1.36
	**Age of sexual maturity**	−2.80	−4.56	−1.08	0.02	2.36
	**Parity**	−0.28	−1.47	0.95	32.74	1.41
	**Reproductive lifespan**	2.36	1.29	3.55	0.00	1.88
	**Hatchling mass**	1.24	0.40	2.12	0.30	2.58
**Frequency**	**Clutch size**	1.17	0.48	1.88	0.06	1.57
	**Clutches per year**	1.20	0.26	2.10	0.54	1.36
	**Age of sexual maturity**	−1.23	−2.19	−0.34	0.48	2.36
	**Parity**	−0.54	−1.20	0.13	5.58	1.41
	**Reproductive lifespan**	1.32	0.69	1.90	0.00	1.88
	**Hatchling mass**	0.35	−0.08	0.82	6.48	2.58

Full parameters for hurdle model predicting US live wildlife trade probability and frequency in reptiles, after iteratively removing variables with the highest VIFs until none were >5 (body mass, *N* = 408, *H*^2^ for binary component = 0.37 [0.15, 0.61], *H*^2^ for poisson component = 0.63 [0.40, 0.82]). Posterior mean = mean β coefficient from posterior distributions, l-95% CI and u-95% CI = lower and upper 95% credible intervals from posterior distributions respectively, % crossing zero = percentage of estimates in the posterior distribution that overlap with zero in the direction opposite to the majority of the distribution, *VIF* variance inflation factors. For ease of interpretation, effects from the binary component of the model are reversed in sign (so that they represent effects on the probability that the outcome variable is 1 or more, rather than 0).

**Table 4:** Life history predictors of US live wildlife trade probability and frequency in amphibians from hurdle models

		Posterior mean	l-95% CI	u-95% CI	% crossing 0	VIF
**Binary**	**Clutch size**	−0.43	−1.78	0.95	26.06	2.58
	**Egg size**	−5.94	−11.38	−0.71	1.60	2.67
	**Age of sexual maturity**	−2.72	−6.42	1.20	8.30	1.32
	**Reproductive lifespan**	4.35	2.19	6.70	0.00	1.12
	**Snout-vent length**	5.51	0.74	9.97	0.68	1.68
**Frequency**	**Clutch size**	0.09	−0.68	1.02	44.46	2.58
	**Egg size**	−1.90	−5.11	1.38	12.68	2.67
	**Age of sexual maturity**	−1.10	−3.26	1.30	16.52	1.32
	**Reproductive lifespan**	1.26	−0.08	2.53	3.74	1.12
	**Snout-vent length**	0.34	−2.25	2.73	39.22	1.68

Full parameters for hurdle model predicting US live wildlife trade probability and frequency in amphibians, including all predictors as none had VIFs>5 (*N* = 132, *H*^2^ for binary component = 0.28 [0.01, 0.63], *H*^2^ for poisson component = 0.20 [0.00, 0.95]). Posterior mean = mean β coefficient from posterior distributions, l-95% CI and u-95% CI = lower and upper 95% credible intervals from posterior distributions respectively, % crossing zero = percentage of estimates in the posterior distribution that overlap with zero in the direction opposite to the majority of the distribution, *VIF* variance inflation factors. For ease of interpretation, effects from the binary component of the model are reversed in sign (so that they represent effects on the probability that the outcome variable is 1 or more, rather than 0).

Within traded species, the probability of introduction increases again with longer reproductive lifespans and some ‘fast’ traits, suggesting that introduced species have a more extreme version of the same life history profile associated with trade (Fig. 1, Tables 5–7). Together with longer reproductive lifespans, introduced mammals have shorter gestational periods and larger litters; introduced reptiles have larger and more frequent clutches; and introduced amphibians have larger clutches (Fig. 1, Tables 5–7), compared with those that are traded but not introduced. Within traded species, body size does not substantially differ between introduced and non-introduced species (Fig. 1, Tables 5–7). When including trade frequency as a predictor of introduction alongside life history traits, we find that trade frequency has a strong positive effect on the probability of introduction in mammals and reptiles, although not amphibians (Supplementary Tables 5–7). Effects of life history traits on introduction largely remain intact when controlling for trade frequency (apart from litter size in mammals, Supplementary Tables 5–7). We find that the probability of introduction increases with the number of occurrence records relative to range size and/or population density, suggesting that highly detectable species are more likely to be recorded as introduced (Supplementary Tables 8–10). However, positive effects of reproductive lifespan on introduction remain when controlling for detectability measures, suggesting that recording biases do not confound the relationship between reproductive lifespan and introduction (Supplementary Tables 11–13).

**Table 5:** Life history predictors of introduction within traded mammals from probit models

	Posterior mean	l-95% CI	u-95% CI	% crossing 0	VIF
**Gestation period**	−1.95	−3.82	0.09	2.16	3.71
**Weaning age**	−0.13	−1.36	1.03	41.98	2.98
**Litter size**	1.77	0.01	3.69	2.28	2.65
**Litters per year**	1.29	−0.45	2.84	6.00	2.84
**Age at first birth**	−0.46	−1.85	1.00	25.30	4.69
**Reproductive lifespan**	2.81	0.88	4.81	0.16	3.38
**Body mass**	0.33	−0.16	0.81	9.38	2.94

Full parameters for model predicting introduction status within mammals traded live by the US, after iteratively removing variables with the highest VIFs until none were >5 (neonatal body mass, *N* = 312, *H*^2^ = 0.54 [0.25, 0.79]). Posterior mean = mean β coefficient from posterior distributions, l-95% CI and u-95% CI = lower and upper 95% credible intervals from posterior distributions respectively, % crossing zero = percentage of estimates in the posterior distribution that overlap with zero in the direction opposite to the majority of the distribution, *VIF* variance inflation factors.

**Table 6:** Life history predictors of introduction within traded reptiles from probit models

	Posterior mean	l-95% CI	u-95% CI	% crossing 0	VIF
**Clutch size**	1.26	0.29	2.17	0.26	1.56
**Clutches per year**	1.43	0.33	2.69	0.94	1.45
**Age of sexual maturity**	0.23	−0.89	1.45	34.96	2.48
**Reproductive lifespan**	1.12	0.37	1.87	0.12	1.81
**Parity**	−0.42	−1.21	0.35	13.56	1.47
**Hatchling mass**	−0.28	−0.79	0.40	17.86	2.57

Full parameters for model predicting introduction within reptiles traded live by the US, after iteratively removing variables with the highest VIFs until none were >5 (body mass, *N* = 285, *H*^2^ = 0.42 [0.05, 0.74]). Posterior mean = mean β coefficient from posterior distributions, l-95% CI and u-95% CI = lower and upper 95% credible intervals from posterior distributions respectively, % crossing zero = percentage of estimates in the posterior distribution that overlap with zero in the direction opposite to the majority of the distribution, *VIF* variance inflation factors.

**Table 7:** Life history predictors of introduction within traded amphibians from probit models

	Posterior mean	l-95% CI	u-95% CI	% crossing 0	VIF
**Clutch size**	0.76	−0.09	1.66	4.04	2.32
**Egg size**	1.59	−2.66	5.99	22.46	2.44
**Age of sexual maturity**	−1.68	−4.79	1.53	14.24	1.50
**Reproductive lifespan**	2.75	0.83	4.90	0.24	1.10
**Snout-vent length**	−0.88	−4.04	2.11	28.36	1.80

Full parameters for model predicting introduction status within amphibians traded live by the US, including all predictors as none had VIFs>5 (*N* = 75, *H*^2^ = 0.30 [0.00, 0.76]). Posterior mean = mean β coefficient from posterior distributions, l-95% CI and u-95% CI = lower and upper 95% credible intervals from posterior distributions respectively, % crossing zero = percentage of estimates in the posterior distribution that overlap with zero in the direction opposite to the majority of the distribution, *VIF* variance inflation factors.

**Fig. 2:**
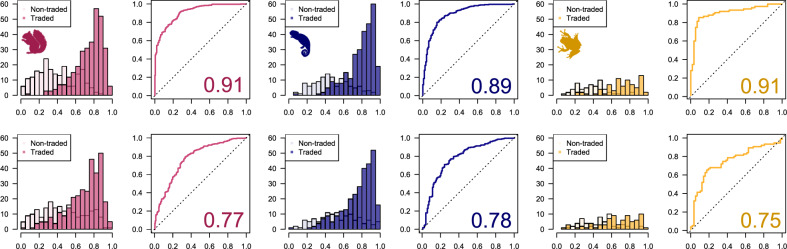
Model classification performance. Classification performance for models based on the US live wildlife trade data across mammal (pink), reptile (blue) and amphibian (gold) species using within-sample predictions (top row) and LOOCV (bottom row). Histograms show distributions of predicted probabilities for traded and non-traded species, while plots show receiver operating characteristic (ROC) curves and areas under the ROC curves (AUC). Silhouettes were obtained from phylopic.org under Public Domain licences.

We use areas under the receiver operating characteristic curve (AUCs) to quantify the ability of the probit models to distinguish traded from non-traded, and introduced from non-introduced species. AUCs vary from 0.5 to 1, where 0.5 indicates the model performs at chance level and 1 indicates perfect discrimination between categories of the outcome variable^39^. We calculate AUCs based both on predictions within the sample as a measure of model fit, and for each species left out of the sample in turn using leave-one-out cross-validation (LOOCV) as a measure of out-of-sample predictive accuracy. Across the three vertebrate classes, AUCs for trade are 0.89 or above for within-sample predictions and 0.75 or above for out-of-sample predictions (Fig. 2, Supplementary Table 14). For introduction, AUCs are at least 0.85 and 0.65 for within- and out-of-sample predictions respectively (Supplementary Table 15). AUCs for introduction are little affected by including trade frequency as an additional predictor (Supplementary Table 16). Both within- and out-of-sample predictions identify very similar lists of species as at high risk of future trade or introduction (Supplementary Data 1). Non-traded species with high out-of-sample predicted probabilities of trade include, for example, golden jackals (*Canis aureus*, 0.86), red-bellied black snakes (*Pseudechis porphyriacus*, 0.98) and Northwestern salamanders (*Ambystoma gracile*, 0.93) (Supplementary Data 1). Non-introduced species in trade with high out-of-sample predicted probabilities of introduction include, for example, Virginia opossums (*Didelphis virginiana*, 0.89), Blanding’s turtle (*Emys blandingii*, 0.81*)* and spotted salamanders (*Ambystoma maculatum*, 0.91) (Supplementary Data 1).

### Pet trade

**Fig. 3:**
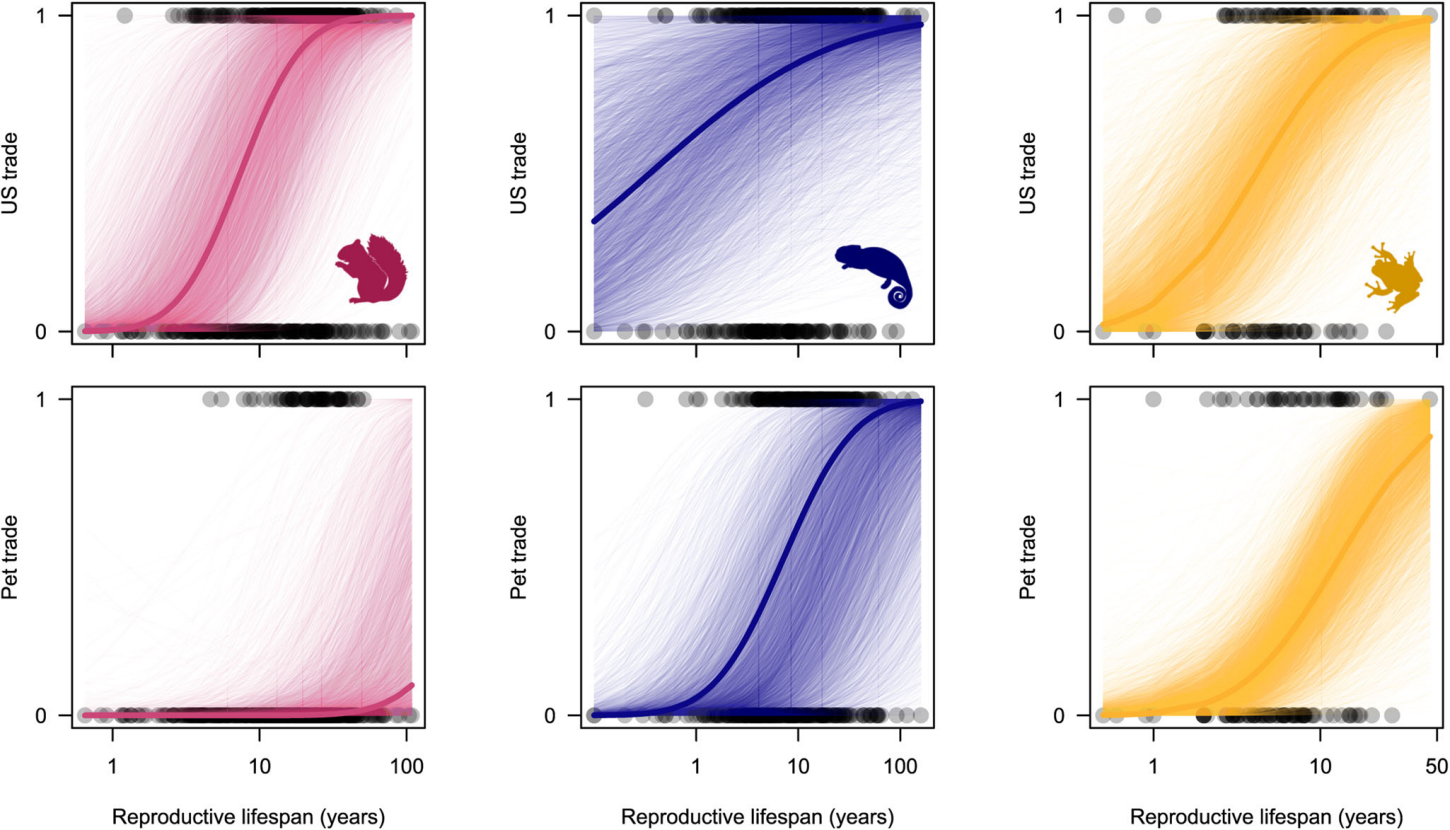
Role of reproductive lifespan in trade. Plots illustrating the relationship between reproductive lifespan and US live trade (top row) and reproductive lifespan and the pet trade (bottom row) for mammals (pink), reptiles (blue) and amphibians (gold). Points represent the raw data values. Faint lines are predicted probit curves from the entire posterior distribution, holding all life history predictors other than reproductive lifespan at the mean. Thick lines represent the mean curve from the posterior distribution, again holding all predictors at the mean apart from reproductive lifespan. Silhouettes were obtained from phylopic.org under Public Domain licences.

Similar to species in the live wildlife trade, pet-traded mammals, reptiles and amphibians consistently have longer reproductive lifespans than non-pet-traded species (Fig. 3, Supplementary Fig. 1, Supplementary Tables 17–19). However, in contrast with the effects of life history traits on general live trade by the US, ‘fast’ life history traits do not predict involvement in pet trade (Supplementary Fig. 1, Supplementary Tables 17–19). Pet-traded mammals have later weaning ages in addition to longer reproductive lifespans (Supplementary Fig. 1, Supplementary Tables 17–19). Therefore, pet-traded species have traits more consistent with ‘slower’ life histories, although life history predictors are generally less consistent across taxa for the pet trade compared with the general live wildlife trade. Interestingly, effects of body mass on trade are not entirely consistent between the general live wildlife trade and the pet trade specifically—for example, while larger mammal species are more likely to be involved in the general live wildlife trade (Table 2), smaller mammal species appear to be targeted for the pet trade, likely for practical reasons (Supplementary Table 17). Long reproductive lifespans also increase the probability of introduction among pet-traded reptiles and amphibians, as do some fast traits in reptiles (clutch size and clutches per year), but otherwise there are no consistent life history predictors of introduction among pet-traded species across the three classes (Supplementary Tables 20–22). Smaller sample sizes for analyses of introduction within pet-traded species compared to US-traded species (Table 1) may partly explain some of these disparities.

AUCs for pet trade models are at least 0.84 and 0.69 for within- and out-of-sample predictions respectively (Supplementary Table 23). Within-sample discriminability for introduction is similar with AUCs of 0.84 or more, while out-of-sample predictive ability is more variable, and in some cases very close to chance levels (Supplementary Table 24). Both within- and out-of-sample predictions flag similar lists of species as having a relatively high risk of future involvement in the pet trade and of introduction within pet-traded species (Supplementary Data 1). However, the predicted probabilities are in fact relatively low in many cases (Supplementary Data 1). For example, in mammals, both sets of predictions identify red-faced spider monkeys (*Ateles paniscus*) among the non-traded species with the highest predicted probabilities of pet trade, but out-of-sample predictions estimate that this species has only a 0.72 probability of involvement in the pet trade (Supplementary Data 1). Similarly, meerkats (*Suricata suricatta*) are consistently identified as among pet-traded species at a relatively high risk of introduction by both sets of predictions, but have an estimated introduction probability of only 0.65 (Supplementary Data 1). We do not run additional introduction models controlling for detectability among pet-traded species due to smaller sample sizes than for US live trade (Table 1).

## Discussion

Life history traits are known to play an important role in the establishment and spread of alien species due to their effects on population growth rates^23–28^, but to our knowledge, no prior study had explained why species selected for introduction have life histories that predispose them towards success at later invasion stages. Here, we addressed this question by disentangling life history biases at the two earliest stages of the invasion pathway: transportation and introduction, with large scale phylogenetic comparative analyses of mammals, reptiles and amphibians. We find that the earliest two invasion stages progressively select for species with increasingly prolific life histories: mammals, reptiles and amphibians in the live wildlife trade have relatively fast life history traits combined with a long reproductive lifespan, while among traded species, those that have been introduced have a more extreme version of this same life history strategy. In mammals and reptiles, species traded most frequently are also most likely to be introduced, consistent with previous studies identifying the wildlife trade as a key introduction pathway in vertebrates^14–17^. Effects of reproductive lifespan on introduction remain when controlling for measures of species detectability, suggesting that observational biases cannot account for these findings. Therefore, our results suggest that ‘introduction biases’ in fact result from a two-stage filtering process early in the invasion pathway, favouring species with distinctive life history profiles. Our findings support the hypotheses that humans prefer to trade in highly fecund species because they are more lucrative for industries involving captive breeding^14,30^, and that species producing numerous offspring over long lifespans have more frequent opportunities to escape and/or are more likely to be deliberately released by owners due to longer-term care requirements^14,15^. The consistency of the life history effects across stages and taxa suggests that human preferences for which species to trade and introduce are not random or arbitrary, but partly determined by widely-shared functional concerns. Given that highly fecund species of mammals, reptiles and amphibians are more likely to establish and spread if released^25,26^, our results suggest that human activities bias the pool of potential invaders towards those most likely to succeed and that many traded species represent a major threat as potential future invaders. While previous analyses identified larger body size as an introduction bias in vertebrates (e.g Refs. ^8,9,11,12,15,22^.), we find that it is in fact associated only with live trade, not with introduction among traded species. This finding highlights the importance of separating the transportation and introduction stages for accurate predictions and well-informed management of future invasions.

Live transportation and release of non-native vertebrates is increasingly driven by the ‘exotic’ pet trade^14–16^. We therefore expected to find the same life history profiles among species in the pet trade as in the live wildlife trade more generally. Apart from long reproductive lifespans, however, pet-traded species do not appear to share the same life history traits as those involved in the general live wildlife trade. Instead, pet-traded species have traits more consistent with a slow life history, such as longer weaning periods in mammals, which should not be advantageous for captive breeding^14,30^. Unlike some prior analyses which have used general live trade as a proxy for the pet trade (e.g. Refs. ^15,19^), our analyses are able to isolate effects specific to the pet trade and so may suggest distinct life history biases associated with the demand for exotic pets. The IUCN records species both in large-scale, industrialised trade for pets as well as those harvested directly from the wild on a smaller scale. While high fecundity is advantageous for the former, slow life histories may be favoured for the latter as a by-product of consumer demand for rarer, threatened species which develop slowly^40^, such as primates. Slowly developing species may also be more appealing as pets due to longer juvenile periods, during which they are less aggressive and perceived as ‘cuter’. However, direct comparison of results based on the US and IUCN trade datasets is difficult given disparities in sample size and representativeness. While the US dataset records imports and exports of all wildlife species, IUCN use and trade descriptions vary in their completeness, likely underestimating the true number of species involved in the pet trade. Pet-traded mammals appear to be particularly under-represented (Table 1), which may be partly due to recording biases: mammals are often traded in small numbers on request, while reptiles and amphibians more openly at markets^41^. In any case, our results suggest that prolific life histories are favoured for large-scale, international trade in live species for purposes that include the pet trade, but are not necessarily associated with demand for pets per se.

Predicting which species are most likely to become invaders in future is a critical issue for conservation and invasive species management, since controlling alien populations once established is often unfeasible^5,6^. Our study shows that life history traits can make above-chance predictions of trade and introduction status, including for species outside of the study sample. Our analyses may therefore help to identify species with life history traits that increase their risk of entering the invasion pathway in the future. For example, analyses based on US wildlife trade data flag golden jackals, red-bellied black snakes and Northwestern salamanders as at high risk of involvement in the live wildlife trade (with out-of-sample predicted probabilities of >0.85). All are plausible candidates for future international trade: since the time of analysis, golden jackals have been reported for sale live at local markets in India^42^, red-bellied black snakes, though venomous, are relatively docile and well-suited to captivity^43^, and many salamander species are already popular in the international pet trade^44^. Our models also highlight several species predicted to be at high risk of future trade that are in fact domesticated and thus already widely traded, but not recorded as such in the US LEMIS dataset, which primarily monitors the wildlife trade. These include for example horses (*Equus caballus*), gayals (*Bos frontalis*) and bantengs (*Bos javanicus*), the latter two being domesticated species that are widely used as livestock and working animals in South and Southeast Asia (Supplementary Data 1). The identification of domesticated species as high risk of trade therefore suggests that our models perform well in identifying species with life histories suitable for human exploitation.

Our models also flag several species traded by the US that have both high (>0.80) out-of-sample predicted probabilities of introduction (Supplementary Data 1) and high invasion potential. For example, Virginia opossums (*Didelphis virginiana*) are closely related to the common opossum (*Didelphis marsupialis*), which has spread extensively in the US following historical introductions beyond its native range^45^. Blanding’s turtle (*Emys blandingii*) is another strong candidate, having already been released within its native range in the US via the pet trade^46^ and belonging to the same family as one of the most notorious invaders in the world: the red-eared slider (*Trachemys scripta elegans)*, with which it shares similar life history characteristics^20,25^. Among amphibians, plausible candidates include spotted salamanders (*Ambystoma maculatum*), which have been exported in large numbers from the US for commercial purposes over the last 20 years^31^. A congener species, the tiger salamander (*Ambystoma tigrinum*), has already been introduced in Europe via the pet trade where it has established alien populations^46^. However, our models also identify some species as at high risk for future trade and introduction that are much less plausible candidates, such as sea turtles (Supplementary Data 1). While sea turtles conform to the life history profile typical of internationally traded and introduced species, with very long reproductive lifespans and large clutches^25^, the high conservation value and inherent difficulty of breeding these species in captivity makes them unlikely candidates. Therefore, predictions are likely to improve in accuracy when further ecological and anthropogenic factors that capture the desirability and feasibility of species for trade and release are factored into future analyses. Such factors, which likely include aesthetic appeal, captive care requirements and associated monetary costs, are poorly understood, partly because they are difficult to quantify, highlighting a need for further research into the motivations of wildlife traders, breeders and owners. A recent study showing that colourful songbird species are targeted for the pet trade^47^, however, suggests that such analyses are feasible and may be usefully applied to other vertebrate groups. While we focus on the effects of life history traits here, the approach we demonstrate has great potential to provide meaningful recommendations for conservation management decisions when expanded to include a wider range of predictors.

Given the ecological and economic harm caused by alien invasive species, understanding why some species are invasive and predicting which species are most likely to be introduced and become invaders in future is a vital issue. The extent to which species traits predict invasion success is a long-standing question, which remains contentious despite numerous comparative analyses^5–7,48,49^. Previous studies have focused on the establishment stage, while those that have considered the introduction stage have typically conflated barriers associated with transportation and release, and few have fully accounted for phylogenetic effects. Here we overcome these limitations by identifying life history predictors of success at the two earliest stages of the invasion pathway, transportation and introduction, using fully phylogenetically informed analyses. We show that mammals, reptiles and amphibians with a highly prolific life history combining fast traits with a long reproductive lifespan are more likely to be transported internationally in the live wildlife trade and to be introduced into non-native regions. These results, together with our earlier findings that highly fecund mammals, reptiles and amphibians are also more likely to establish and spread once introduced^25,26^, demonstrate that human activities favour the release of species with life histories that predispose them towards becoming abundant, widespread invaders. Taken together, our studies show that species’ life histories are a key predictor of success across the entire invasion pathway and that barriers at each stage of the pathway—transportation, introduction, establishment and spread—progressively select for the most prolific species. While species with slower life histories may be at greater risk of demand for exotic pets, only those with life histories suited to captive breeding are likely to be favoured for industrial-scale pet trade. Many of our models are able to predict the trade and introduction status of species outside the sample at well above chance levels, and thus allow us to identify several plausible candidates for future trade and introduction, including some species with high invasion potential. Our approach therefore offers a valuable framework for both understanding why some species are invasive and identifying which are most likely to become invasive in future.

## Methods

### Data collection

We obtained trade data from two different sources: the United States Fish and Wildlife Service (USFWS) Law Enforcement Management Information System (LEMIS)^31^ and the International Union for Conservation of Nature (IUCN) Red List^32^. We used the former to obtain data on the live wildlife trade in general and the latter for data on the pet trade specifically. We then matched trade data with our previously compiled global scale datasets of life history traits and introductions in mammals, reptiles and amphibians^25,26^.

We obtained data on the US live wildlife trade from LEMIS by a Freedom of Information Act Request on 12/08/2019. We requested summary data on all US imports and exports of wildlife across all available years (1999-2019) and all trade purposes, including information on species identities and shipment contents (e.g. live individuals, meat, skins, etc.). For each species, we summed the total number of recorded shipments of live individuals (including individuals that died in transit, and live eggs) as a measure of trade frequency. We classified species as in trade if there was at least one shipment of live individuals recorded in the LEMIS database, and as not traded otherwise. The LEMIS dataset is geographically limited to trade by the US, and therefore may not capture the full diversity of species involved in the wildlife trade. For example, the LEMIS database may be missing some species involved in the substantial trade in live wildlife between South–East Asian countries^50^. However, the US represents one of the most dominant players in the global market for live wildlife^16^, and by summing both imports and exports we capture demand for species in countries beyond the US to some extent. Supplementary Fig. 2 illustrates the frequency of trade between the US and countries represented in the US LEMIS dataset. LEMIS data should be considered a minimum estimate of the diversity of species involved in the wildlife trade since they mostly record only legal trade (although confiscated shipments are recorded), and shipments are sometimes not identified to the species level^16,51–53^. The LEMIS database also contains some mis-spelled and incorrectly identified species due to human input errors^52^. To minimise the effect of misidentified shipments on our species level classifications of US trade status, we discarded all LEMIS records that were not identified to the species level (i.e. those identified using genus, common or generic names only), and manually checked the LEMIS data for synonyms and alternate spellings when we could not automatically match any records in LEMIS with species in our life history datasets. Species classified as traded on the basis of a single recorded live shipment in LEMIS are most vulnerable to species level misclassification due to misidentified shipments. The vast majority of traded species have multiple shipments recorded in LEMIS (259/312 [83%] of traded mammals, 265/285 [93%] of traded reptiles and 72/75 [96%] of traded amphibians), reducing the potential impact of shipment level misidentification over the reliability of species level trade classifications. However, to investigate the robustness of our findings to possible errors in species identification in LEMIS, we re-ran our key analyses excluding species classified as traded on the basis of a single live shipment. We found qualitatively the same effects of life history traits on the probability of trade when removing these species as in our full sample (Supplementary Tables 25–27). Despite its limitations, LEMIS is an invaluable resource for identifying broad scale trends in the wildlife trade since few other countries maintain such detailed records, and it is the only large-scale international trade dataset that includes both CITES- and non-CITES-listed species^16,41^. Including non-CITES listed species in our analyses is important because CITES-listed species represent only a small minority of those in trade^14^ and are likely to be a biased sample in terms of life history traits, since species vulnerable to extinction typically have slower life histories^40^.

We obtained separate data on the pet trade from the IUCN Red List. The IUCN has assessed the vast majority of mammal, reptile and amphibian species (91%, 79% and 86% respectively^54^). Here, we classified a species as involved in the pet trade if the IUCN species account included at least one clear description of involvement in the pet trade. Otherwise, we considered a species as not involved in the pet trade. Although LEMIS records the purpose of trade, it uses broad categories (e.g. ‘Commercial’, ‘Personal’, ‘Breeding in captivity’), none of which refers specifically to nor necessarily equates to trade for pets. Therefore, we sought this additional data on the pet trade from the IUCN Red List instead of following the approach of some previous studies which have used LEMIS data as a proxy for the pet trade (e.g. Refs. ^15,19^). In contrast, the IUCN Red List contains clear textual descriptions of use and trade for many species, allowing us to identify which species are traded specifically for pets^32^. The IUCN data has further complementary strengths compared with LEMIS in that it is global in scope and includes both legal and illegal trade. We obtained data from the IUCN Red List by manually searching the binomial name of each species in our samples and consulting the ‘Threats’ and ‘Use and Trade’ sections of the species accounts. We classified species as in the pet trade if the information clearly stated this was the case (e.g. “It has been recorded in the pet trade”, “This species appears in the international pet trade”). We discounted descriptions where the information was uncertain (e.g. the species is described as “probably” or “possibly” traded for pets). We did not count as pets those species that the IUCN categorises as used for “Pets/display animals, horticulture” but which are used only for zoos or captive display, such as beluga whales (*Delphinapterus leucas*). All species described as pets by the IUCN are ‘exotic’, i.e. those without a long history of domestication^14^, since the IUCN does not list domesticated species.

We matched trade data with our previously published global scale datasets on life history traits and introductions^25,26^. Internationally traded species may or not be released in the wild outside their native range: some may remain in the confines of captivity (e.g. in zoos or kept by private owners). We defined a species as introduced if there was at least one reliable record of its release, by humans, into the wild outside of its native range, either accidentally or intentionally^25,26^. We included only species with complete data for the same life history traits as used in our prior analyses (mammals: body mass, gestation period, weaning age, neonatal body mass, litter size, litters per year, age at first reproduction and reproductive lifespan; reptiles: body mass, hatchling mass, clutch size, clutches per year, age of sexual maturity, reproductive lifespan and parity; amphibians: snout-vent length, egg size, clutch size, age of sexual maturity and reproductive lifespan) to facilitate direct comparisons with previous results and to allow us to account for covariation between life history traits^55^. Species with complete life history data represent 7.8%, 3.5% and 1.6% of the total estimated number of species of mammals, reptiles and amphibians respectively^56–58^. These samples are not random as they over-represent orders containing many species of interest and utility to humans (e.g. ungulates, primates, crocodilians) (Supplementary Tables 28–30). However, these biases are unlikely to undermine our results since we examine life history effects on trade and introduction within these samples. Trade and introduction data do not necessarily cover the same time periods: the US dataset covers only the years 1999-present and the IUCN descriptions also typically refer to recent trade. In contrast, our introduction dataset includes both historical and recent introductions^25,26^. Therefore, the goal of our analyses is not to test causal hypotheses on the direct relationship between trade and introduction but rather to investigate whether the same life history traits predispose species towards both trade and introduction across diverse taxa, locations and circumstances. When combining the datasets and phylogenies^59–63^, we resolved species name mis-matches by referring to taxonomic information from the IUCN Red List^32^, the Global Biodiversity Information Facility (GBIF^33^) and the Integrated Taxonomic Information System (ITIS^64^). Table 1 summarises final sample sizes and Supplementary Table 1 the degree of overlap between the trade datasets. Most species in the pet trade are also in the general live wildlife trade, but many more species are traded by the US for general purposes than are involved in the pet trade specifically.

Finally, we obtained data for a proxy measure of species detectability in order to control for a potential confounding effect on relationships between life history traits and introduction: larger bodied and longer-lived species may be more likely to be recorded by human observers when introduced compared with smaller and shorter-lived species. We obtained data on species occurrence records, geographic range size and population density, assuming that highly detectable species will have a disproportionately large number of recorded observations than expected based on the size of their geographic ranges and average population densities, following similar approaches by e.g. Refs. ^65,66^. We obtained occurrence records from the Global Biodiversity Information Facility (GBIF^33^) via the R package *rgbif*^67^ selecting only records resulting from human observation. We obtained range sizes (in decimal degrees squared) from the IUCN Red List^32^ and processed them for analysis using functions from the *rgdal* package^68^, excluding areas of uncertain presence (i.e. limiting range to presence code 1, ‘extant’). We obtained population density estimates from the TetraDENSITY database (version 1^34^), a global database of population density estimates for terrestrial vertebrates. Most species in the TetraDENSITY dataset are represented by estimates from multiple different studies (median = 3, range 1–408). We collapsed density estimates to the species level by taking the median value across studies, including all estimates regardless of sampling method to maximise sample size, and converting all units to individuals/km^2^ to ensure comparability.

### Statistical analyses

To investigate relationships between life history traits and trade, we run models treating US or pet trade as the outcome variable and life history traits as the predictors. For all analyses, all life history variables were included in the same models to account for covariation among life history traits^55^. For US trade, where data on trade frequency are available, we run models both in which trade is treated as a binary variable (traded vs. not traded) and as a count variable (frequency of live shipments, including zero values), while for the pet trade, we have no data on trade frequency and so we treat pet trade as a binary variable only. To investigate the effects of life history traits on introduction, we run models in which introduction is the outcome variable and life history traits are the predictors. In introduction models, we only include traded species (running separate models for the set of species in US trade and the set of species in the pet trade). This approach allows us to disentangle effects associated with trade and introduction and thus identify at which stage(s) life history biases emerge. We also run introduction models in which frequency of US trade is included as an additional predictor alongside life history traits, anticipating that highly traded species are more likely to be introduced. Finally, to investigate possible confounding effects of species detectability on relationships between life history traits and introduction, we investigate effects of number of observations, geographic range size and, where sample sizes allowed, population density on the probability of introduction. If highly detectable species are more likely to be recorded as introduced, we expect to find a positive effect of the number of observations (while accounting for geographic range size and population density) on the probability of introduction. If this effect confounds relationships between body mass/lifespan and introduction, the effect of these life history traits on the probability of introduction should disappear when detectability measures are included in the models alongside life history traits. All analyses were conducted using the R statistical programming environment (Version 4.2.0^69^). Plots were coloured using palettes from the *viridis* package^70^.

To estimate effects of predictor variables, we fit generalized linear mixed models (GLMMs) using Markov chain Monte-Carlo (MCMC) estimation, implemented in the MCMCglmm package^35,36^. For analyses with binary outcome variables (traded vs. not traded, introduced vs. not introduced) we fit probit models, while for analyses with US trade frequency as the outcome variable we fit hurdle models. Hurdle models estimate two latent variables: the probability that the outcome is zero (on the logit scale), and the probability of the outcome modelled as a Poisson distribution for non-zero values^71^. This method therefore allows us to estimate effects of life history traits on the probability and frequency of trade in the same model. While the binary component of a hurdle model estimates the probability that outcomes are zero, when reporting results we reverse the sign of coefficients from the binary model for ease of interpretation, so that effects can be interpreted as the probability that the outcome is *not* zero. Therefore, here predictors with consistent effects on the probability and frequency of trade in hurdle models will have the same sign (so that if, for example, litter size has a positive effect on both the probability and frequency of trade, both coefficients for litter size from the hurdle model will be positive).

Datasets comprising biological measures from multiple related species violate the fundamental statistical assumption that observations are independent of one another, since closely related species are more phenotypically similar than expected by chance due to their shared evolutionary history^72^. To account for the non-independence of species due to shared ancestry, we included a phylogenetic random effect in all models, represented by a variance-covariance (VCV) matrix derived from the phylogeny. The off-diagonal elements of the VCV matrix contain the amount of shared evolutionary history for each pair of species^35,37,38^ based on the branch lengths of the phylogeny (here proportional to time)^59–63^. This approach allows us to estimate phylogenetic signal using the heritability (*H*^2^) parameter, which measures the proportion of total variance in the latent variable attributable to the phylogeny^35,37,38^. Heritability is interpreted in the same way as Pagel’s λ in phylogenetic generalized least squares regression^35,37,38,72^. Specifically, phylogenetic signal is constrained between 0, indicating no phylogenetic effect so that species can be treated as independent, and 1, indicating that similarity between species is directly proportional to their amount of shared evolutionary history^35,38,72^. As hurdle models estimate two latent variables, for each hurdle model we report two heritability estimates, one for the binary and one for the Poisson component. All continuous independent variables were log-10 transformed due to positively skewed distributions. Although GLMMs do not require normally distributed predictor variables, log-transforming positively skewed life history predictors in phylogenetic comparative analyses allows us to model life history evolution on proportional rather than absolute scales. This is important as it facilitates biologically meaningful comparisons between species across large scales of life history variation^73^. Further, log-transforming positively skewed predictors helps to meet assumptions of the underlying Brownian motion model of evolutionary change, which assumes that phenotypic change along branches of the phylogeny is normally distributed^74^.

We calculated variance inflation factors (VIFs) using functions from the *car* R package^75^ to check for multicollinearity between predictor variables. Where any model reported a variance inflation factor of 5 or above, indicating potentially problematic levels of collinearity^76^, we re-ran the model removing the variable with the highest VIF iteratively until all the remaining variables had VIFs of <5. We also illustrate the correlations between the life history variables included in our models in Supplementary Figs. 3–5, which suggest evidence for both classic fast-slow life history trade-offs (e.g. smaller, less frequent litters in larger, longer-lived mammal species) and more complex patterns (e.g. larger clutches in larger-bodied, longer-lived reptile species). For each model, we report the mean estimates from posterior distributions for all parameters, and the percentage of fixed effect parameter estimates crossing zero in the direction opposite to the majority of estimates, as a measure of the strength of evidence for individual fixed effects in a specific direction. We used default, diffuse normal prior probability distributions for the fixed effects (mean = 0, variance = 10^10^). For the phylogenetic random effect in probit models, we used a chi-squared prior distribution which better approximates a uniform prior compared with more commonly used inverse-Gamma priors^37^ (with *V* = 1, *ν* = 1000, *αμ* = 0, *αV* = 1^71^). The residual variance is fixed to 1 since models with binary dependent variables do not provide sufficient information to estimate residual variance (following^37^). For the binary component of the hurdle model, we used the same chi-squared prior for the phylogenetic random effect and fixed prior for the residual variance as we used in the probit model. For the Poisson component of the hurdle model, we used commonly implemented inverse-Wishart priors (with *V* = 1 and *ν* = 0.002, equivalent to inverse-gamma distributions with shape and scale = 0.001^71^) for the phylogenetic random effect and the residual variance. By modelling residual variance separately, MCMCglmm accounts for over-dispersion in the distribution of the non-zero response values^71^, which is common in count data. We ran each model for a sufficient number of iterations to obtain effective sample sizes of at least 1000 for all parameters (5,010,000 iterations, with a burn-in period of 10,000 iterations, sampling every 1000 generations). Model convergence was also confirmed by visual examination of posterior distributions and chain plots.

Finally, we assessed the ability of our models to predict the probability of trade and introduction for species within and outside the sample, based on both fixed effects (life history traits and body mass) and the phylogenetic random effect. For out-of-sample predictions, we used leave-one-out cross-validation (LOOCV), i.e. we re-ran the model excluding each species in turn, obtained predictions for the missing species and compared these with the observed values. For both types of predictions,  we calculated the area under the curve (AUC) of the receiver operating characteristic (ROC) curve as a measure of classification performance, using the cvAUC package^77^. AUC values indicate the probability that a randomly drawn positive observation (in this case, a species that is traded or introduced) has a predicted value that is greater than a randomly drawn negative observation (i.e. a species that is not traded or introduced)^39^. AUCs vary from 0.5 to 1, where 0.5 indicates that the model predictions are no better than random guesses, and 1 indicates perfect distinction (i.e. positive observations always have greater predicted probabilities than negative observations)^39^. Next, we used predicted values from the model to identify species not listed as traded or introduced in our datasets, but with high predicted probabilities of trade or introduction which may indicate high risk of future trade or introduction. To do so, we extracted predicted probabilities from models and identified non-traded or non-introduced species with high predicted probabilities for each vertebrate class. LOOCV required re-running each of our models as many times as the N species in the sample, which necessitated shorter MCMC chains to avoid impractically long run-times. In supplementary analyses, we show that predictions do not differ between models based on short versus long chains (Supplementary Table 31).

### Reporting summary

Further information on research design is available in the Nature Portfolio Reporting Summary linked to this article.

## Supplementary information

Supplementary Information

Description of Additional Supplementary Files

Supplementary Data 1

Reporting Summary

## Data Availability

All data required to replicate the results of this study have been deposited in the Dryad repository and are available at the following link: 10.5061/dryad.8cz8w9gvb.

## Code availability

All R scripts required to replicate the results of this study are available from the Dryad repository at the following link: 10.5061/dryad.8cz8w9gvb.
