# Supplementary material for: Human activities favour prolific life histories in both traded and introduced vertebrates
Source: Nat Commun. 2023 Jan 17;14:262. doi: 10.1038/s41467-022-35765-6 (PMC9845321; doi:10.1038/s41467-022-35765-6)
Supplement: Supplementary file 1 — Supplementary Information [file 41467_2022_35765_MOESM1_ESM.pdf]

Supplementary Information for: Street S.E., Gutiérrez J.S., Allen W.L. & Capellini I. "Human activities favour prolific life histories in both traded and introduced species"

SUPPLEMENTARY TABLES

Table 1: overlap between trade datasets

Class	Total species	N traded (US)	N pet traded	N traded (both)
Mammals	518	312	67	47
Reptiles	408	285	183	157
Amphibians	132	75	48	37

Number of species in the US live wildlife trade, in the pet trade, and in both types of trade.

Table 2: life history predictors of US trade in mammals from probit model

	Posterior mean	l-95% CI	u-95% CI	% crossing 0	VIF
**Gestation period**	0.03	-1.58	1.66	48.44	4.34
**Weaning age**	-0.04	-1.12	0.90	47.62	2.82
**Litter size**	2.10	0.57	3.57	0.14	2.78
**Litters per year**	0.95	-0.25	2.29	7.02	2.62
**Reproductive lifespan**	2.99	1.77	4.36	0.00	3.28
**Body mass**	0.44	0.02	0.84	1.08	3.29

Full parameters for probit model predicting US trade status in mammals, after iteratively removing variables with the highest VIFs until none were >5 (neonatal body mass and age at first birth, N=518, H^2=0.59 [0.33, 0.79]). Posterior mean = mean β coefficient from posterior distributions, l-95% CI and u-95% CI = lower and upper 95% credible intervals from posterior distributions respectively, % crossing zero = percentage of estimates in the posterior distribution that overlap with zero in the direction opposite to the majority of the distribution, VIF = variance inflation factors.

Table 3: life history predictors of US trade in reptiles from probit model

	Posterior mean	l-95% CI	u-95% CI	% crossing 0	VIF
**Clutch size**	1.21	0.31	2.04	0.22	1.57
**Clutches per year**	0.88	-0.15	1.85	4.38	1.36
**Age of sexual maturity**	-1.13	-2.15	-0.03	1.86	2.36
**Parity**	0.11	-0.64	0.80	38.02	1.41
**Reproductive lifespan**	0.72	0.08	1.39	1.64	1.88
**Hatchling mass**	0.65	0.06	1.20	1.24	2.58

Full parameters for probit model predicting US trade status in reptiles, after iteratively removing variables with the highest VIFs until none were >5 (body mass, N=408, H^2=0.47 [0.21, 0.73]). Posterior mean = mean β coefficient from posterior distributions, l-95% CI and u-95% CI = lower and upper 95% credible intervals from posterior distributions respectively, % crossing zero = percentage of estimates in the posterior distribution that overlap with zero in the direction opposite to the majority of the distribution, VIF = variance inflation factors.

Table 4: life history predictors of US trade in amphibians from probit model

	Posterior mean	l-95% CI	u-95% CI	% crossing 0	VIF
**Clutch size**	-0.33	-1.19	0.52	21.04	2.58
**Egg size**	-2.81	-6.12	0.45	4.42	2.67
**Age of sexual maturity**	-1.26	-3.68	1.13	13.70	1.32
**Reproductive lifespan**	2.18	0.89	3.56	0.12	1.12
**Snout-vent length**	3.92	0.70	6.92	0.30	1.68

Full parameters for probit model predicting US trade status in amphibians, including all predictor variables as none had VIFs >5 (N=132, H^2=0.43 [0.02, 0.80]). Posterior mean = mean β coefficient from posterior distributions, l-95% CI and u-95% CI = lower and upper 95% credible intervals from posterior distributions respectively, % crossing zero = percentage of estimates in the posterior distribution that overlap with zero in the direction opposite to the majority of the distribution, VIF = variance inflation factors.

Table 5: trade frequency and life history predictors of introduction in mammals from probit model

	Posterior mean	l-95% CI	u-95% CI	% crossing 0	VIF
**Trade frequency**	1.02	0.51	1.55	0.02	1.15
**Gestation period**	-1.93	-4.02	-0.06	2.34	3.77
**Weaning age**	-0.42	-1.63	0.71	23.80	3.04
**Litter size**	1.05	-0.75	3.02	13.44	2.80
**Litters per year**	1.02	-0.60	2.64	10.76	2.87
**Age at first birth**	-0.49	-1.91	0.81	25.12	4.69
**Reproductive lifespan**	2.26	0.25	4.28	1.10	3.50
**Body mass**	0.32	-0.21	0.81	10.80	2.98

Full parameters for probit model predicting introduction status from trade frequency and life history traits within mammal species traded live by the US, after iteratively removing variables with the highest VIFs until none were >5 (neonatal body mass, N=312, H^2=0.55 [0.26, 0.79]). Posterior mean = mean β coefficient from posterior distributions, l-95% CI and u-95% CI = lower and upper 95% credible intervals from posterior distributions respectively, % crossing zero = percentage of estimates in the posterior distribution that overlap with zero in the direction opposite to the majority of the distribution, VIF = variance inflation factors.

Table 6: trade frequency and life history predictors of introduction in reptiles from probit model

	Posterior mean	l-95% CI	u-95% CI	% crossing 0	VIF
**Trade frequency**	0.74	0.35	1.14	0.00	1.15
**Clutch size**	0.99	0.16	1.92	1.12	1.62
**Clutches per year**	1.20	0.06	2.30	1.62	1.51
**Age of sexual maturity**	0.61	-0.54	1.76	14.04	2.53
**Parity**	-0.26	-1.01	0.43	23.70	1.47
**Reproductive lifespan**	0.79	0.07	1.56	1.70	1.92
**Hatchling mass**	-0.37	-0.88	0.22	9.14	2.57

Full parameters for probit model predicting introduction status from trade frequency and life history traits within reptile species traded live by the US, after iteratively removing variables with the highest VIFs until none were >5 (body mass, N=285, H^2=0.34 [0.01, 0.73]). Posterior mean = mean β coefficient from posterior distributions, l-95% CI and u-95% CI = lower and upper 95% credible intervals from posterior distributions respectively, % crossing zero = percentage of estimates in the posterior distribution that overlap with zero in the direction opposite to the majority of the distribution, VIF = variance inflation factors.

Table 7: trade frequency and life history predictors of introduction in amphibians from probit model

	Posterior mean	l-95% CI	u-95% CI	% crossing 0	VIF
**Trade frequency**	-0.08	-0.91	0.75	42.48	1.18
**Clutch size**	0.77	-0.08	1.68	3.96	2.33
**Egg size**	1.58	-2.71	5.97	22.84	2.52
**Age of sexual maturity**	-1.72	-4.98	1.52	14.88	1.53
**Reproductive lifespan**	2.84	0.73	4.97	0.32	1.18
**Snout-vent length**	-0.94	-4.16	2.33	27.68	1.80

Full parameters for probit model predicting introduction status from trade frequency and life history traits within amphibian species traded live by the US, including all predictor variables as none had VIFs >5 (N=75, H^2=0.32 [0.00, 0.77]). Posterior mean = mean β coefficient from posterior distributions, l-95% CI and u-95% CI = lower and upper 95% credible intervals from posterior distributions respectively, % crossing zero = percentage of estimates in the posterior distribution that overlap with zero in the direction opposite to the majority of the distribution, VIF = variance inflation factors.

Table 8: effect of detectability on introduction in mammals from probit model

	Posterior mean	l-95% CI	u-95% CI	% crossing 0	VIF
**N observations**	0.85	0.44	1.29	0.00	1.61
**Range size**	0.03	-0.43	0.51	46.50	1.62
**Density**	0.32	0.05	0.62	0.82	1.01

Full parameters for probit model predicting introduction status within mammals traded by the US from number of human observations recorded in the GBIF database, range size and population density (N=162, H^2=0.19 [0.00, 0.60]). Posterior mean = mean β coefficient from posterior distributions, l-95% CI and u-95% CI = lower and upper 95% credible intervals from posterior distributions respectively, % crossing zero = percentage of estimates in the posterior distribution that overlap with zero in the direction opposite to the majority of the distribution, VIF = variance inflation factors.

Table 9: effect of detectability on introduction in reptiles from probit models

	Posterior mean	l-95% CI	u-95% CI	% crossing 0	VIF
**N observations**	0.82	-0.01	1.66	1.72	1.15
**Range size**	0.65	-0.16	1.47	4.92	1.16
**Density**	-0.05	-0.37	0.30	39.00	1.03

Full parameters for probit model predicting introduction status within reptiles traded by the US from number of human observations recorded in the GBIF database, range size and population density (N=77, H^2=0.34 [0.00, 0.75]). Posterior mean = mean β coefficient from posterior distributions, l-95% CI and u-95% CI = lower and upper 95% credible intervals from posterior distributions respectively, % crossing zero = percentage of estimates in the posterior distribution that overlap with zero in the direction opposite to the majority of the distribution, VIF = variance inflation factors.

Table 10: effect of detectability on introduction in amphibians from probit models

	Posterior mean	l-95% CI	u-95% CI	% crossing 0	VIF
**N observations**	0.95	0.19	1.77	0.38	2.12
**Range size**	-0.24	-0.95	0.46	24.54	2.12

Full parameters for probit model predicting introduction status within amphibians traded by the US from number of human observations recorded in the GBIF database and range size (N=75, H^2=0.32 [0.00, 0.77]). Population density could not be included due to insufficient sample size (density estimates were available for only 14 species with data on N observations and range size). Posterior mean = mean β coefficient from posterior distributions, l-95% CI and u-95% CI = lower and upper 95% credible intervals from posterior distributions respectively, % crossing zero = percentage of estimates in the posterior distribution that overlap with zero in the direction opposite to the majority of the distribution, VIF = variance inflation factors.

Table 11: effect of detectability & life history traits on introduction in mammals from probit model

	Posterior mean	l-95% CI	u-95% CI	% crossing 0	VIF
**N observations**	0.95	0.47	1.42	0.00	1.72
**Range size**	0.00	-0.55	0.50	48.28	1.86
**Density**	0.48	0.09	0.86	0.90	2.42
**Weaning age**	-0.26	-1.83	1.34	38.56	3.91
**Litter size**	-0.85	-2.85	1.02	19.70	2.22
**Litters per year**	0.74	-1.26	2.85	24.00	3.48
**Age at first birth**	-1.99	-3.87	-0.14	1.84	4.91
**Reproductive lifespan**	2.24	-0.13	4.54	2.74	2.93
**Body mass**	0.40	-0.22	1.00	10.24	3.81

Full parameters for probit model predicting introduction status within mammals traded by the US from number of human observations recorded in the GBIF database, range size, population density and life history traits, after iteratively removing variables with the highest VIFs until none were >5 (neonatal body mass and gestation time, N=162, H^2=0.17 [0.00, 0.63]). Posterior mean = mean β coefficient from posterior distributions, l-95% CI and u-95% CI = lower and upper 95% credible intervals from posterior distributions respectively, % crossing zero = percentage of estimates in the posterior distribution that overlap with zero in the direction opposite to the majority of the distribution, VIF = variance inflation factors.

Table 12: effect of detectability & life history traits on introduction in reptiles from probit model

	Posterior mean	l-95% CI	u-95% CI	% crossing 0	VIF
**N observations**	0.48	-0.65	1.55	19.66	1.60
**Range size**	1.72	0.57	2.93	0.10	1.34
**Density**	-0.09	-0.60	0.37	35.40	1.98
**Clutch size**	4.51	1.68	7.61	0.00	2.07
**Clutches per year**	7.43	3.30	11.82	0.00	2.68
**Age of sexual maturity**	0.58	-2.94	4.30	36.54	2.88
**Parity**	-0.46	-2.69	1.65	34.14	1.57
**Reproductive lifespan**	4.57	2.15	7.33	0.00	2.03
**Hatchling mass**	-0.76	-2.32	0.77	16.14	3.32

Full parameters for probit model predicting introduction status within reptiles traded by the US from number of human observations recorded in the GBIF database, range size, population density and life history traits, after iteratively removing variables with the highest VIFs until none were >5 (adult body mass, N=77, H^2=0.39 [0.00, 0.80]). Posterior mean = mean β coefficient from posterior distributions, l-95% CI and u-95% CI = lower and upper 95% credible intervals from posterior distributions respectively, % crossing zero = percentage of estimates in the posterior distribution that overlap with zero in the direction opposite to the majority of the distribution, VIF = variance inflation factors.

Table 13: effect of detectability & life history traits on introduction in amphibians from probit model

	Posterior mean	l-95% CI	u-95% CI	% crossing 0	VIF
**N observations**	1.21	0.28	2.25	0.32	2.27
**Range size**	-0.52	-1.47	0.36	11.92	2.75
**Clutch size**	0.46	-0.66	1.68	22.10	3.76
**Egg size**	1.18	-3.55	6.11	31.34	2.77
**Age of sexual maturity**	-1.07	-4.97	2.17	27.48	1.55
**Reproductive lifespan**	2.76	0.61	4.94	0.40	1.13
**Snout-vent length**	0.71	-3.49	4.92	37.34	2.57

Full parameters for probit model predicting introduction status within amphibians traded by the US from number of human observations recorded in the GBIF database, range size and life history traits (N=75, H^2=0.36 [0.00, 0.81]). Population density could not be included due to insufficient sample size (density estimates were available for only 14 amphibian species with data on N observations and range size). Posterior mean = mean β coefficient from posterior distributions, l-95% CI and u-95% CI = lower and upper 95% credible intervals from posterior distributions respectively, % crossing zero = percentage of estimates in the posterior distribution that overlap with zero in the direction opposite to the majority of the distribution, VIF = variance inflation factors.

Table 14: AUC values for US trade models

Class	Prediction type	AUC
Mammals	Within-sample	0.91
	LOOCV	0.77
Reptiles	Within-sample	0.89
	LOOCV	0.78
Amphibians	Within-sample	0.91
	LOOCV	0.75

Area-under-the-curve (AUC) values for models predicting US trade status from life history traits, for both within-sample and leave-one-out cross-validation (LOOCV) predictions, across mammals, reptiles and amphibians.

Table 15: AUC values for introduction models (within US traded species)

Class	Prediction type	AUC
Mammals	Within-sample	0.89
	LOOCV	0.71
Reptiles	Within-sample	0.86
	LOOCV	0.69
Amphibians	Within-sample	0.85
	LOOCV	0.65

Area-under-the-curve (AUC) values for models predicting introduction status from life history traits within species traded live by the US, for both within-sample and leave-one-out cross-validation (LOOCV) predictions, across mammals, reptiles and amphibians.

Table 16: AUC values for introduction models including trade frequency (within US traded species)

Class	Prediction type	AUC
Mammals	Within-sample	0.90
	LOOCV	0.73
Reptiles	Within-sample	0.84
	LOOCV	0.70
Amphibians	Within-sample	0.85
	LOOCV	0.64

Area-under-the-curve (AUC) values for models predicting introduction status from life history traits and trade frequency within species traded live by the US, for both within-sample and leave-one-out cross-validation (LOOCV) predictions, across mammals, reptiles and amphibians.

Table 17: **life history predictors of pet trade in mammals from probit model**

	Posterior mean	l-95% CI	u-95% CI	% crossing 0	VIF
**Gestation period**	0.98	-1.46	3.65	21.62	4.34
**Weaning age**	1.83	0.25	3.64	1.26	2.82
**Litter size**	0.40	-1.90	2.63	36.08	2.78
**Litters per year**	0.40	-1.69	2.27	34.56	2.62
**Reproductive lifespan**	2.49	0.44	4.80	1.20	3.28
**Body mass**	-1.15	-1.89	-0.48	0.04	3.29

Full parameters for probit model predicting pet trade status in mammals, after iteratively removing variables with the highest VIFs until none were >5 (neonatal body mass and age at first birth, N=518, H^2=0.73 [0.50, 0.88]). Posterior mean = mean β coefficient from posterior distributions, l-95% CI and u-95% CI = lower and upper 95% credible intervals from posterior distributions respectively, % crossing zero = percentage of estimates in the posterior distribution that overlap with zero in the direction opposite to the majority of the distribution, VIF = variance inflation factors.

Table 18: life history predictors of pet trade in reptiles from probit model

	Posterior mean	l-95% CI	u-95% CI	% crossing 0	VIF
**Clutch size**	-0.76	-1.75	0.29	6.44	1.57
**Clutches per year**	0.79	-0.43	2.04	9.64	1.36
**Age of sexual maturity**	-0.78	-2.04	0.46	10.86	2.36
**Parity**	-0.74	-1.71	0.19	5.74	1.41
**Reproductive lifespan**	1.86	1.04	2.72	0.00	1.88
**Hatchling mass**	1.33	0.66	2.02	0.02	2.58

Full parameters for probit model predicting pet trade status in reptiles, after iteratively removing variables with the highest VIFs until none were >5 (body mass, N=408, H^2=0.69 [0.49, 0.84]). Posterior mean = mean β coefficient from posterior distributions, l-95% CI and u-95% CI = lower and upper 95% credible intervals from posterior distributions respectively, % crossing zero = percentage of estimates in the posterior distribution that overlap with zero in the direction opposite to the majority of the distribution, VIF = variance inflation factors.

Table 19: life history predictors of pet trade in amphibians from probit model

	Posterior mean	l-95% CI	u-95% CI	% crossing 0	VIF
**Clutch size**	-0.39	-1.06	0.31	13.56	2.58
**Egg size**	-0.99	-3.70	2.10	24.74	2.67
**Age of sexual maturity**	-1.43	-3.54	0.59	8.10	1.32
**Reproductive lifespan**	2.21	0.93	3.49	0.02	1.12
**Snout-vent length**	0.88	-1.67	3.21	23.20	1.68

Full parameters for probit model predicting pet trade status in amphibians, including all predictor variables as none had VIFs >5 (N=132, H^2=0.27 [0.00, 0.67]). Posterior mean = mean β coefficient from posterior distributions, l-95% CI and u-95% CI = lower and upper 95% credible intervals from posterior distributions respectively, % crossing zero = percentage of estimates in the posterior distribution that overlap with zero in the direction opposite to the majority of the distribution, VIF = variance inflation factors.

Table 20: life history predictors of introduction within pet-traded mammals from probit model

	Posterior mean	l-95% CI	u-95% CI	% crossing 0	VIF
**Gestation period**	-1.82	-7.68	3.46	26.28	4.99
**Weaning age**	1.16	-2.46	4.49	24.68	3.74
**Litter size**	2.47	-2.92	7.64	17.34	4.49
**Litters per year**	2.49	-1.79	6.92	12.30	2.91
**Age at first birth**	-0.89	-5.07	3.04	33.60	4.37
**Reproductive lifespan**	3.04	-1.70	8.39	11.28	2.95
**Body mass**	-0.14	-1.52	1.18	41.54	1.77

Full parameters for probit model predicting introduction status within mammals in the pet trade, after iteratively removing variables with the highest VIFs until none were >5 (neonatal body mass, gestation length, N=67, H^2=0.46 [0.01, 0.83]). Posterior mean = mean β coefficient from posterior distributions, l-95% CI and u-95% CI = lower and upper 95% credible intervals from posterior distributions respectively, % crossing zero = percentage of estimates in the posterior distribution that overlap with zero in the direction opposite to the majority of the distribution, VIF = variance inflation factors.

Table 21: life history predictors of introduction within pet-traded reptiles from probit model

	Posterior mean	l-95% CI	u-95% CI	% crossing 0	VIF
**Clutch size**	2.75	1.52	4.15	0.00	1.37
**Clutches per year**	2.32	0.79	3.84	0.06	1.60
**Age of sexual maturity**	-0.62	-2.25	0.87	22.00	2.53
**Parity**	0.00	-1.16	1.12	48.10	1.33
**Reproductive lifespan**	1.38	0.13	2.72	1.62	1.92
**Hatchling mass**	-0.53	-1.33	2.24	8.76	2.21

Full parameters for probit model predicting introduction status within reptiles in the pet trade, after iteratively removing variables with the highest VIFs until none were >5 (adult body mass, N=183, H^2=0.48 [0.07, 0.78]). Posterior mean = mean β coefficient from posterior distributions, l-95% CI and u-95% CI = lower and upper 95% credible intervals from posterior distributions respectively, % crossing zero = percentage of estimates in the posterior distribution that overlap with zero in the direction opposite to the majority of the distribution, VIF = variance inflation factors.

Table 22: life history predictors of introduction within pet-traded amphibians from probit model

	Posterior mean	l-95% CI	u-95% CI	% crossing 0	VIF
**Clutch size**	0.57	-0.54	1.84	17.90	2.69
**Egg size**	0.15	-5.61	5.78	46.92	2.73
**Age of sexual maturity**	-2.87	-7.22	0.94	7.06	1.40
**Reproductive lifespan**	2.76	0.06	5.71	1.36	1.23
**Snout-vent length**	0.83	-4.39	6.32	38.28	2.67

Full parameters for model predicting introduction status within amphibians in the pet trade (N=48, H^2=0.33 [0.00, 0.80]). Posterior mean = mean β coefficient from posterior distributions, l-95% CI and u-95% CI = lower and upper 95% credible intervals from posterior distributions respectively, % crossing zero = percentage of estimates in the posterior distribution that overlap with zero in the direction opposite to the majority of the distribution, VIF = variance inflation factors.

Table 23: AUC values for pet trade models

Class	Prediction type	AUC
Mammals	Within-sample	0.97
	LOOCV	0.85
Reptiles	Within-sample	0.95
	LOOCV	0.83
Amphibians	Within-sample	0.84
	LOOCV	0.69

Area-under-the-curve (AUC) values for models predicting pet trade status from life history traits, for both within-sample and leave-one-out cross-validation (LOOCV) predictions, across mammals, reptiles and amphibians.

Table 24: AUC values for introduction models (within pet-traded species)

Class	Prediction type	AUC
Mammals	Within-sample	0.90
	LOOCV	0.52
Reptiles	Within-sample	0.90
	LOOCV	0.76
Amphibians	Within-sample	0.84
	LOOCV	0.52

Area-under-the-curve (AUC) values for models predicting introduction status from life history traits within pet-traded species, for both within-sample and leave-one-out cross-validation (LOOCV) predictions, across mammals, reptiles and amphibians

Table 25: life history predictors of US trade in mammals from probit model, excluding species classified on the basis of a single shipment

	Posterior mean	l-95% CI	u-95% CI	% crossing 0	VIF
**Gestation period**	-0.21	-2.03	1.54	40.56	4.22
**Weaning age**	0.20	-0.85	1.34	35.56	2.92
**Litter size**	2.62	1.02	4.36	0.08	2.73
**Litters per year**	1.24	-0.27	2.59	4.80	2.60
**Reproductive lifespan**	4.07	2.48	5.73	0.00	3.26
**Body mass**	0.44	-0.03	0.89	2.24	3.27

Full results of probit model predicting US trade status in mammals after removing species classified as in trade on the basis of a single record (N=465, H^2=0.66 [0.43, 0.83]). Posterior mean = mean β coefficient from posterior distributions, l-95% CI and u-95% CI = lower and upper 95% credible intervals from posterior distributions respectively, % crossing zero = percentage of estimates in the posterior distribution that overlap with zero in the direction opposite to the majority of the distribution, VIF = variance inflation factors.

Table 26: life history predictors of US trade in reptiles from probit model, excluding species classified on the basis of a single shipment

	Posterior mean	l-95% CI	u-95% CI	% crossing 0	VIF
**Clutch size**	1.35	0.42	2.32	0.16	1.55
**Clutches per year**	1.26	0.14	2.44	1.46	1.37
**Age of sexual maturity**	-1.23	-2.44	-0.11	1.92	2.36
**Parity**	0.07	-0.70	0.91	42.22	1.39
**Reproductive lifespan**	1.15	0.36	1.93	0.18	1.93
**Hatchling mass**	0.66	0.09	1.33	1.66	2.57

Full results of probit model predicting US trade status in reptiles after removing species classified as in trade on the basis of a single record (N=388, H^2=0.56 [0.29, 0.78]). Posterior mean = mean β coefficient from posterior distributions, l-95% CI and u-95% CI = lower and upper 95% credible intervals from posterior distributions respectively, % crossing zero = percentage of estimates in the posterior distribution that overlap with zero in the direction opposite to the majority of the distribution, VIF = variance inflation factors.

Table 27: life history predictors of US trade in amphibians from probit model, excluding species classified on the basis of a single shipment

	Posterior mean	l-95% CI	u-95% CI	% crossing 0	VIF
**Clutch size**	-0.28	-1.17	0.60	25.44	2.58
**Egg size**	-3.19	-6.60	0.30	3.10	2.67
**Age of sexual maturity**	-1.06	-3.58	1.35	19.60	1.32
**Reproductive lifespan**	2.41	0.90	3.74	0.00	1.12
**Snout-vent-length**	4.23	0.79	7.45	0.30	1.68

Full results of probit model predicting US trade status in amphibians after removing species classified as in trade on the basis of a single record (N=129, H^2=0.47 [0.05, 0.81]). Posterior mean = mean β coefficient from posterior distributions, l-95% CI and u-95% CI = lower and upper 95% credible intervals from posterior distributions respectively, % crossing zero = percentage of estimates in the posterior distribution that overlap with zero in the direction opposite to the majority of the distribution, VIF = variance inflation factors.

Table 28: results of permutation tests investigating sampling bias in mammals

Order	N species	Complete LH	Expected
Afrosoricida	55	4	4 [1-9]
Artiodactyla	366	99*	28 [19-39]
Carnivora	315	102*	25 [16-34]
Chiroptera	1456	19*	114 [96-132]
Cingulata	22	3	2 [1-5]
Dasyuromorphia	78	1*	6 [2-11]
Didelphimorphia	125	4	10 [4-16]
Diprotodontia	158	19	12 [6-19]
Eulipotyphla	575	18*	45 [33-58]
Hyracoidea	6	2	1 [1-2]
Lagomorpha	107	13	8 [3-14]
Macroscelidea	20	3	2 [1-4]
Monotremata	5	1	1 [1-2]
Peramelemorphia	30	2	2 [1-6]
Perissodactyla	18	9*	2 [1-4]
Pilosa	17	4	2 [1-4]
Primates	526	74*	41 [30-53]
Proboscidea	3	2	1 [1-2]
Rodentia	2683	135*	210 [189-231]
Scandentia	23	1	2 [1-5]
Sirenia	5	2	1 [1-2]
Tubulidentata	1	1	1 [1-1]

Comparison of the total number of mammal species in each order with those represented in the set of species with complete life history data. 'N species' = total number of mammal species in each order recognized by the Mammal Diversity Database[1], 'Complete LH' = number of species in each order with complete life history data in our samples, expected = median and 95% interval (2.5th and 97.5th percentiles) based on 10,000 random samples. Asterisks indicate orders in which the observed number falls above or below the expected 95% interval, indicating orders over- or under-represented in the life history dataset.

Table 29: results of permutation tests investigating sampling bias in reptiles

Order	N species	Complete LH	Expected
Crocodilia	26	19*	1 [1-3]
Rhynchocephalia	1	1	1 [1-1]
Squamata	11182	355*	394 [387-401]
Testudines	361	33*	13 [6-20]

Comparison of the total number of reptile species in each order with those represented in the set of species with complete life history data. 'N species' = total number of species in each order recognized by the Reptile Database[2], 'Complete LH' = number of species in each order with complete life history data in our samples, expected = median and 95% interval (2.5th and 97.5th percentiles) based on 10,000 random samples. Asterisks indicate orders in which the observed number falls above or below the expected 95% interval, indicating orders over- or under-represented in the life history dataset.

Table 30: results of permutation tests investigating sampling bias in amphibians

Order	N species	Complete LH	Expected
Anura	7401	89*	117 [109-123]
Caudata	765	43*	12 [6-19]

Comparison of the total number of amphibian species in each order with those represented in the set of species with complete life history data. 'N species' = total number of species recognized by AmphibiaWeb[3], 'Complete LH' = number of species in each order with complete life history data in our samples, expected = median and 95% interval (2.5th and 97.5th percentiles) based on 10,000 random samples. Asterisks indicate orders in which the observed number falls above or below the expected 95% interval, indicating orders over- or under-represented in the life history dataset.

Table 31: comparison of shorter chains used in LOOCV predictions with full chains

Binomial	Common name	Prediction (short)	Prediction (long)
Tachyglossus aculeatus	Short-beaked echidna	0.66	0.65
Didelphis marsupialis	Common opossum	0.55	0.55
Perameles gunnii	Eastern barred bandicoot	0.41	0.41
Phascolarctos cinereus	Koala	0.66	0.66
Lasiorhinus latifrons	Southern hairy-nosed wombat	0.74	0.73
Trichosurus vulpecula	Common brushtail possum	0.56	0.56
Petaurus breviceps	Sugar glider	0.59	0.58
Potorous tridactylus	Long-nosed potoroo	0.60	0.60
Bettongia gaimardi	Tasmanian bettong	0.61	0.59
Bettongia lesueur	Burrowing bettong	0.51	0.51

Comparison of LOOCV predicted probabilities of US trade for a sample of 10 species based on 'short' and 'long' MCMC chains. 'Short' chains were run for 13,000 iterations, with a burn-in period of 3,000 iterations, sampling every 10 generations. 'Long' chains consisted of 5,010,000 iterations, with a burn-in of 10,000 iterations, sampling every 1000 generations.

SUPPLEMENTARY FIGURES

SI Figure 1

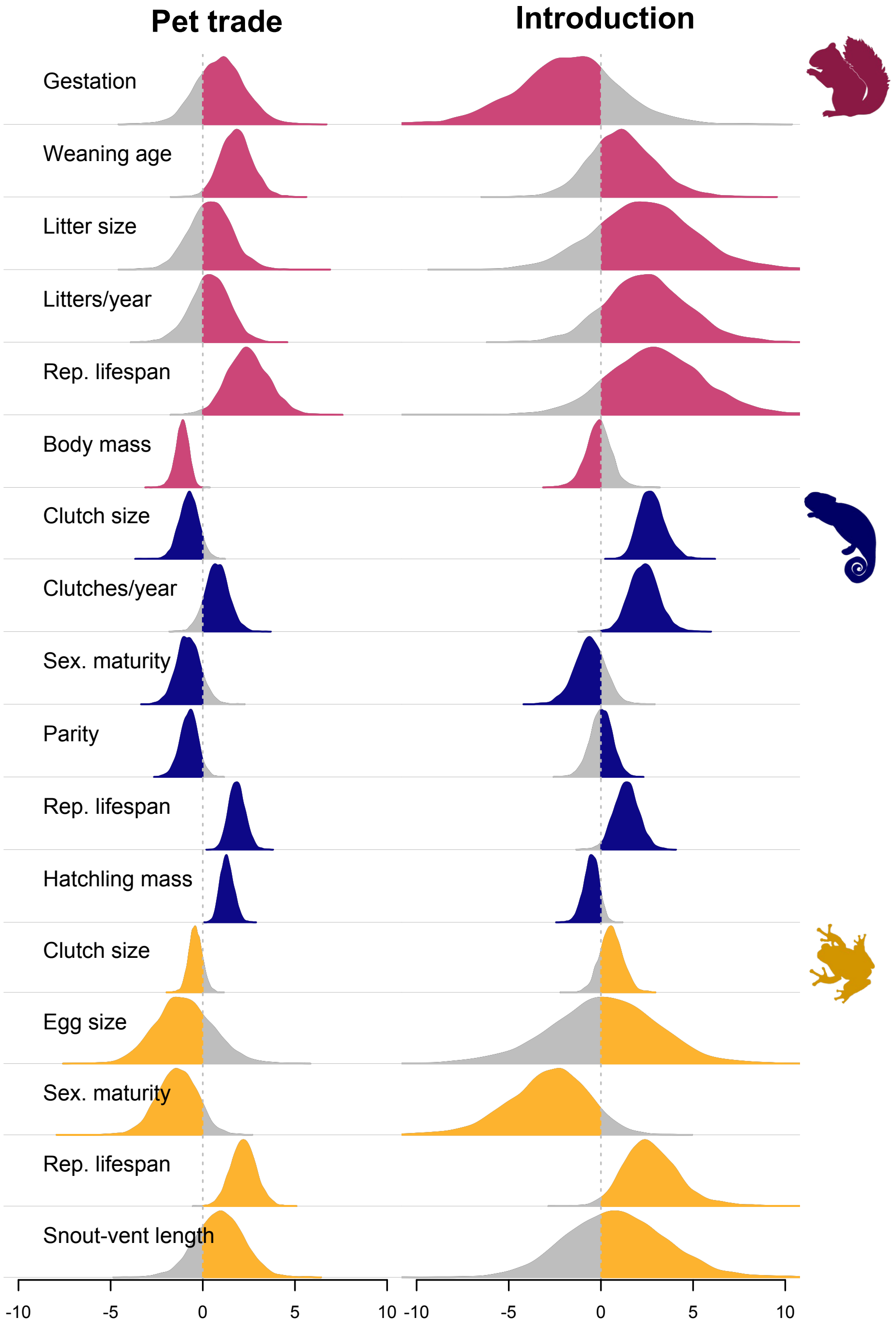

Posterior distributions of fixed effect estimates for models based on the IUCN pet-trade data, for both trade and introduction (within pet-traded species) across mammals (pink), reptiles (blue) and amphibians (gold). Silhouettes were obtained from phylopic.org under Public Domain licenses.

SI Figure 2

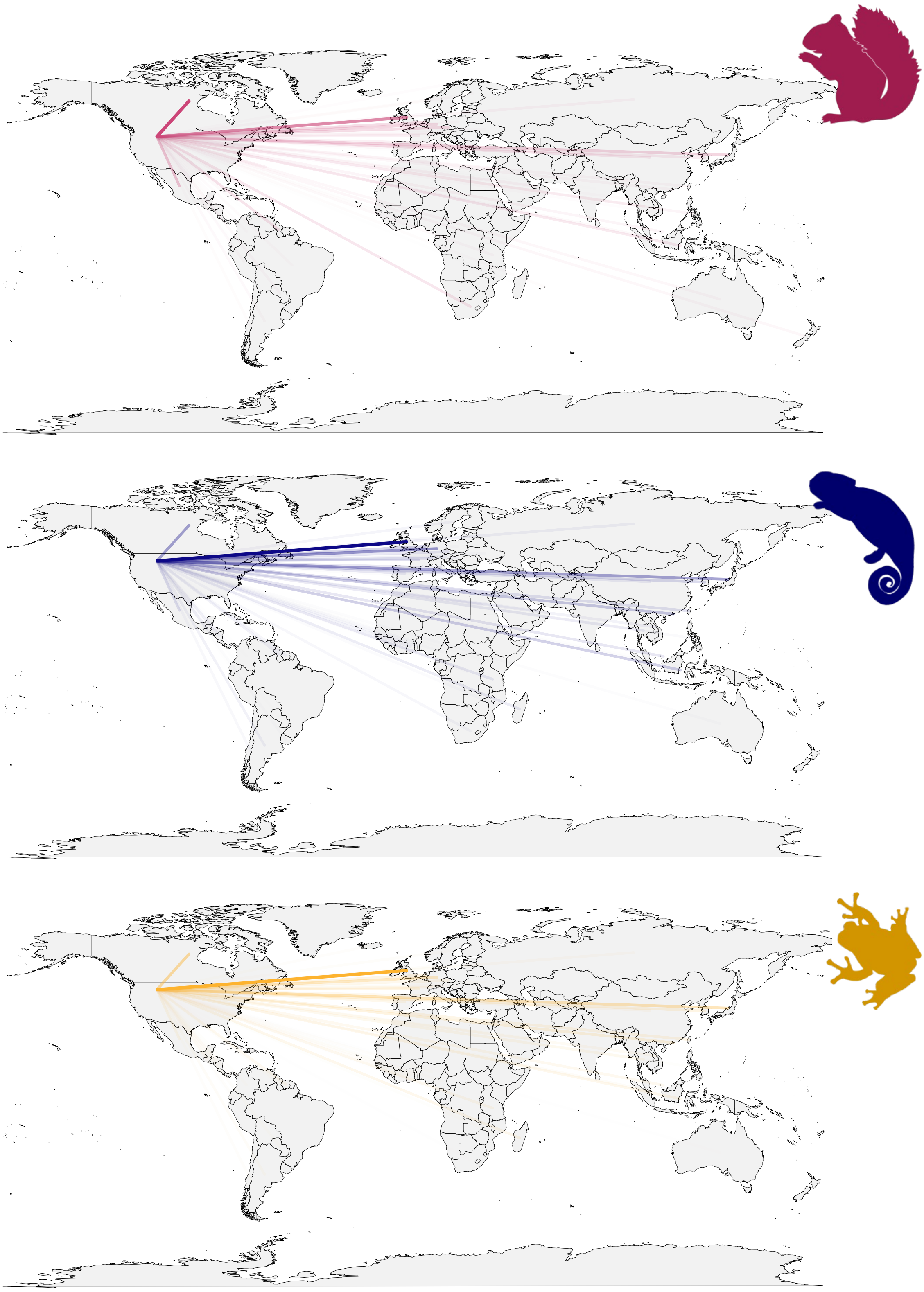

Figure illustrating the frequency of live trade between the USA and other countries recorded in the LEMIS dataset (1999-2019) for mammals (pink), reptiles (blue) and amphibians (gold). Darker/thicker lines indicate higher numbers of shipments (summed exports and imports of live animals). This figure was created using functions from the rworldmap[4], rgeos[5], network[6] and maps[7] R packages. Silhouettes were obtained from phylopic.org under Public Domain licenses.

SI Figure 3

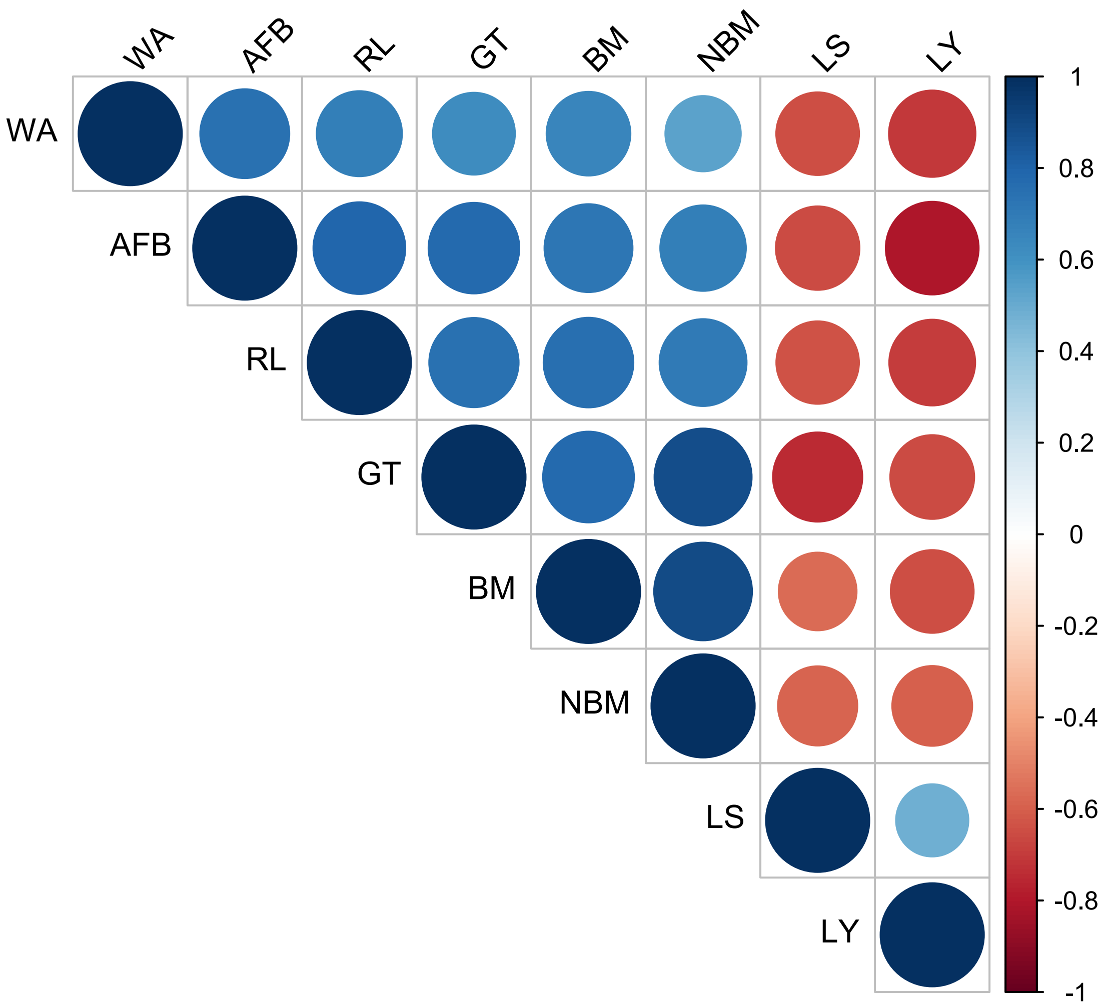

Correlogram illustrating relationships (Pearson's correlations) between life history traits in mammals. WA=weaning age, AFB=age of first birth, RL=reproductive lifespan, GT=gestation time, BM=body mass, NBM=neonatal body mass, LS=litter size, LY=litters per year. Circle size and colour indicate the strength and direction of correlations. This figure was created using the corrplot package[8].

SI Figure 4

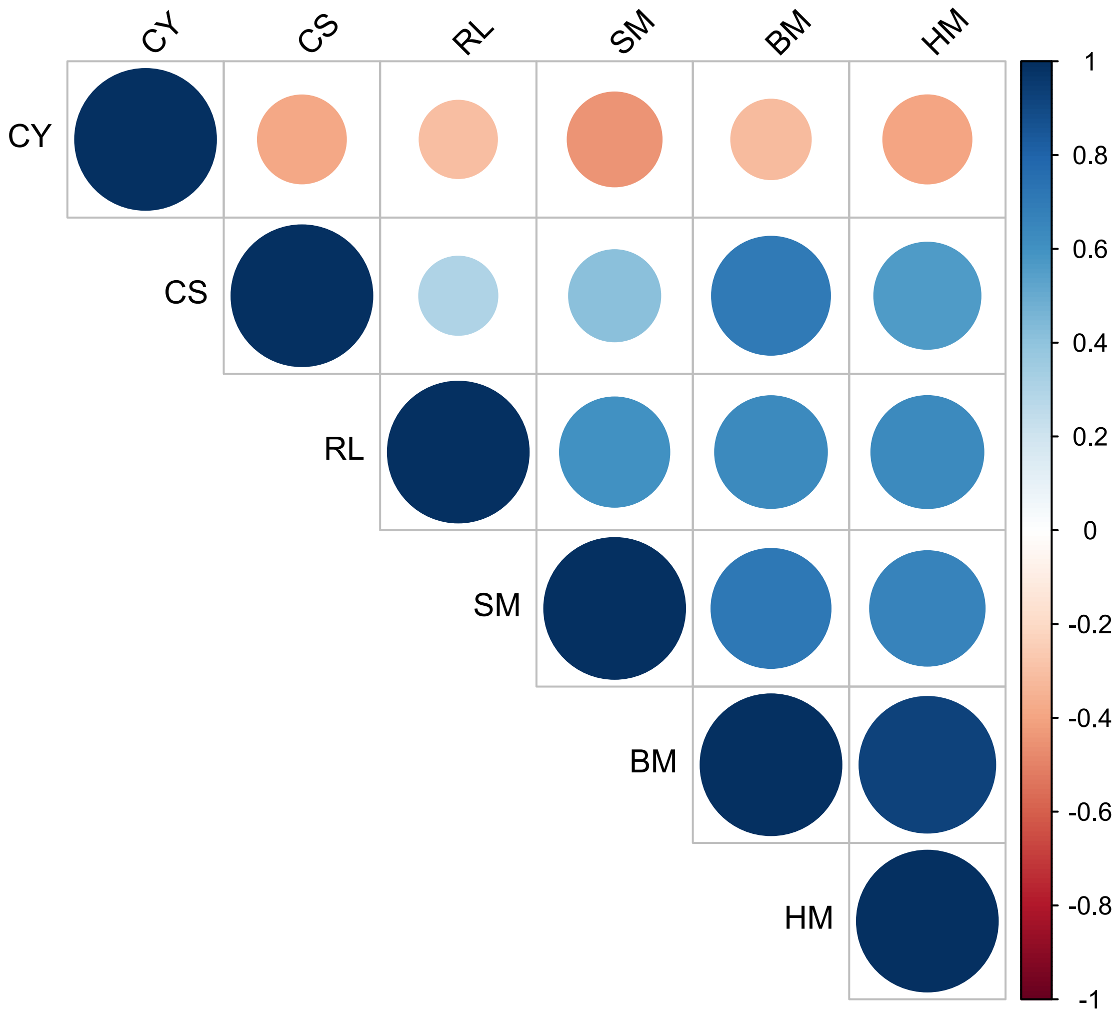

Correlogram illustrating relationships (Pearson's correlations) between life history traits in reptiles. CY=clutches per year, CS=clutch size, RL=reproductive lifespan, SM=age of sexual maturity, BM=body mass, HM=hatchling mass. Circle size and colour indicate the strength and direction of correlations. This figure was created using the corrplot package[8].

SI Figure 5

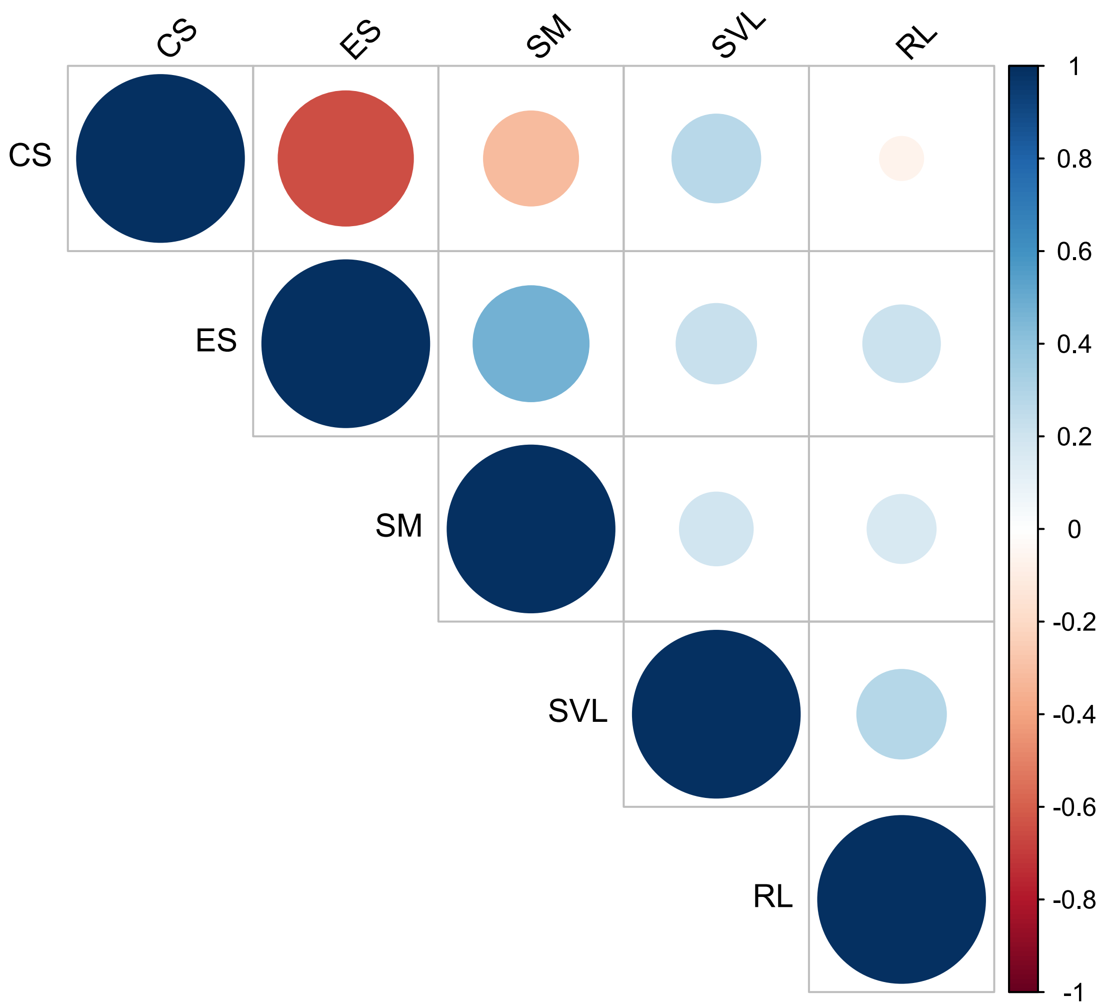

Correlogram illustrating relationships (Pearson's correlations) between life history traits in amphibians. CS=clutch size, ES=egg size, SM=age of sexual maturity, SVL=snout-vent length, RL=reproductive lifespan. Circle size and colour indicate the strength and direction of correlations. This figure was created using the corrplot package[8].

Bibliography

1. American Society of Mammalogists. Mammal Diversity Database (Version 1.10). (2022). Available at: http://doi.org/10.5281/zenodo.4139818. (Accessed: 19th December 2022)
2. Uetz, P., Freed, P., Aguilar, R. & Hošek, J. The Reptile Database. (2021). Available at: http://www.reptile-database.org/. (Accessed: 15th September 2021)
3. University of California, Berkeley, CA, U. AmphibiaWeb. (2021). Available at: https://amphibiaweb.org. (Accessed: 15th September 2021)
4. South, A. rworldmap: A New R package for Mapping Global Data. *R J.* **3**, 35–43 (2011). Available at: http://journal.r-project.org/archive/2011-1/RJournal_2011-1_South.pdf
5. Bivand, R. & Rundel, C. rgeos: Interface to Geometry Engine - Open Source ('GEOS'). (2021). Available at: https://CRAN.R-project.org/package=rgeos
6. Butts, C. network: a Package for Managing Relational Data in R. *J. Stat. Softw.* **24**, (2008).
7. Brownrigg, R. maps: Draw Geographical Maps. (2021). Available at: https://CRAN.R-project.org/package=network
8. Wei, T. & Simko, V. R package 'corrplot': Visualization of a Correlation Matrix. (2021). Available at: https://github.com/taiyun/corrplot
