# Supplementary material for: Human activities favour prolific life histories in both traded and introduced vertebrates
Source: Nat Commun. 2023 Jan 17;14:262. doi: 10.1038/s41467-022-35765-6 (PMC9845321; doi:10.1038/s41467-022-35765-6)
Supplement: Supplementary file 2 — Description of Additional Supplementary Files [file 41467_2022_35765_MOESM2_ESM.pdf]

Description of Additional Supplementary Files

File Name: Supplementary Data 1
Description: Predicted values for trade and introduction status from life history models.
