# Supplementary material for: Human activities favour prolific life histories in both traded and introduced vertebrates
Source: Nat Commun. 2023 Jan 17;14:262. doi: 10.1038/s41467-022-35765-6 (PMC9845321; doi:10.1038/s41467-022-35765-6)
Supplement: Supplementary file 4 — Reporting Summary [file 41467_2022_35765_MOESM4_ESM.pdf]

nature portfolio

Corresponding author(s): Sally Street, Isabella Capellini

Last updated by author(s): Dec 9, 2022

# Reporting Summary

Nature Portfolio wishes to improve the reproducibility of the work that we publish. This form provides structure for consistency and transparency in reporting. For further information on Nature Portfolio policies, see our Editorial Policies and the Editorial Policy Checklist.

## Statistics

For all statistical analyses, confirm that the following items are present in the figure legend, table legend, main text, or Methods section.

| n/a | Confirmed | |
|---|---|---|
| ☐ | ☒ | The exact sample size (*n*) for each experimental group/condition, given as a discrete number and unit of measurement |
| ☒ | ☐ | A statement on whether measurements were taken from distinct samples or whether the same sample was measured repeatedly |
| ☒ | ☐ | The statistical test(s) used AND whether they are one- or two-sided<br>*Only common tests should be described solely by name; describe more complex techniques in the Methods section.* |
| ☐ | ☒ | A description of all covariates tested |
| ☐ | ☒ | A description of any assumptions or corrections, such as tests of normality and adjustment for multiple comparisons |
| ☐ | ☒ | A full description of the statistical parameters including central tendency (e.g. means) or other basic estimates (e.g. regression coefficient) AND variation (e.g. standard deviation) or associated estimates of uncertainty (e.g. confidence intervals) |
| ☒ | ☐ | For null hypothesis testing, the test statistic (e.g. *F*, *t*, *r*) with confidence intervals, effect sizes, degrees of freedom and *P* value noted<br>*Give P values as exact values whenever suitable.* |
| ☐ | ☒ | For Bayesian analysis, information on the choice of priors and Markov chain Monte Carlo settings |
| ☒ | ☐ | For hierarchical and complex designs, identification of the appropriate level for tests and full reporting of outcomes |
| ☐ | ☒ | Estimates of effect sizes (e.g. Cohen's *d*, Pearson's *r*), indicating how they were calculated |

*Our web collection on statistics for biologists contains articles on many of the points above.*

## Software and code

Policy information about availability of computer code

| | |
|---|---|
| Data collection | Datasets were merged and processed for analysis primarily using base functions in the R statistical programming environment (version 4.2.0). We obtained species occurrence records from the GBIF database using the rgbif package (version 3.7.2). Species range maps were downloaded from the IUCN Red List and processed for analysis using the rgdal package (version 1.5-30). |
| Data analysis | Data were analysed using the MCMCglmm (version 2.34), car (version 3.1-1), cvUAC (version 1.1.4) packages, with additional custom functions written by the first author (S.E.S.). SI Figure 2 was created using functions from the R packages rworldmap (version 1.3-6), rgeos (version 0.5-9), network (version 1.13.0.1) and maps (version 3.4.0). SI Figures 3-5 were created using functions from the corrplot package (version 0.90). Images were coloured using palettes from the viridis R package (version 0.6.1). All R code used for the analyses, including custom functions, is available from the Dryad repository at the following link: https://doi.org/10.5061/dryad.8cz8w9gvb |

For manuscripts utilizing custom algorithms or software that are central to the research but not yet described in published literature, software must be made available to editors and reviewers. We strongly encourage code deposition in a community repository (e.g. GitHub). See the Nature Portfolio guidelines for submitting code & software for further information.

## Data

Policy information about availability of data

All manuscripts must include a data availability statement. This statement should provide the following information, where applicable:

- Accession codes, unique identifiers, or web links for publicly available datasets
- A description of any restrictions on data availability
- For clinical datasets or third party data, please ensure that the statement adheres to our policy

All data required to replicate the results of this study have been deposited in the Dryad repository and are available at the following link: https://doi.org/10.5061/dryad.8cz8w9gvb. Wildlife trade data were obtained from the United States Fish and Wildlife Service Law Enforcement Management Information System database by freedom of information request. Pet trade classifications were based on species descriptions in the International Union for the Conservation of Nature's Red List (https://www.iucnredlist.org/). We obtained data on introduction status and life history traits from two prior studies (Allen et al. 2017, Ecology Letters; Capellini et al. 2015, Ecology Letters) available from the Dryad repository at the following links: https://doi.org/10.5061/dryad.2d7b0; https://doi.org/10.5061/dryad.rk4jp). Species occurrence records were obtained from the Global Biodiversity Information Facility (https://www.gbif.org/) using the occ_count function from the rgbif package (version 3.7.2). Polygons for species geographic ranges were downloaded from the IUCN Red List (https://www.iucnredlist.org/resources/spatial-data-download). Population density estimates were obtained from the TetraDENSITY database (version 1), available from the following link: https://figshare.com/articles/dataset/TetraDENSITY_Population_Density_dataset/5371633. Finally, we obtained estimates of the total number of mammal, reptile and amphibian species from the Mammal Diversity Database (version 1.10, http://doi.org/10.5281/zenodo.4139818), the Reptile Database (http://www.reptile-database.org/) and Amphibiaweb (https://amphibiaweb.org).

## Human research participants

Policy information about studies involving human research participants and Sex and Gender in Research.

| | |
|---|---|
| Reporting on sex and gender | NA: study involved no human research participants |
| Population characteristics | NA: study involved no human research participants |
| Recruitment | NA: study involved no human research participants |
| Ethics oversight | NA: study involved no human research participants |

Note that full information on the approval of the study protocol must also be provided in the manuscript.

# Field-specific reporting

Please select the one below that is the best fit for your research. If you are not sure, read the appropriate sections before making your selection.

☐ Life sciences ☐ Behavioural & social sciences ☒ Ecological, evolutionary & environmental sciences

For a reference copy of the document with all sections, see nature.com/documents/nr-reporting-summary-flat.pdf

# Ecological, evolutionary & environmental sciences study design

All studies must disclose on these points even when the disclosure is negative.

| | |
|---|---|
| Study description | This study uses phylogenetic comparative methods to investigate life history traits as predictors of trade and introduction status across species from three vertebrate classes: mammals, reptiles and amphibians. The analyses are conducted at the species-level. |
| Research sample | The research sample contains mammal, reptile and amphibian species with complete data for a set of key life history variables, which we compiled for two previous publications (Allen et al. 2017, Ecology Letters 20: 222-230, https://doi.org/10.1111/ele.12728; Capellini et al. 2015, Ecology Letters 18: 1099-1107, https://doi.org/10.1111/ele.12493). We chose to use species with complete data on multiple life history traits because it was important to account for correlations between life history predictors in our analyses, and because this facilitates comparison with our previous results on the role of life history traits in success at later stages of the invasion pathway. As we acknowledge in our paper, these samples are not necessarily representative of the full diversity of mammal, reptile and amphibian species – orders containing many species of interest and utility to humans are over-represented, such as ungulates, primates and crocodilians. However, we identify consistent effects of life history traits on trade and introduction within these samples and have no reason to suspect that these patterns would not generalise to other, less well studied species. |
| Sampling strategy | Sample sizes (n=518 for mammals, n=408 for reptiles and n=132 for amphibians) were determined by the availability of life history data (please see above for further details). These were the maximum sample sizes we could use while including only species with complete life history data in the analyses. For the vast majority of our analyses, we have sufficient statistical power based on the 'one in ten' rule (i.e. 10 or more observations per statistical parameter estimated), and in the few cases where sample sizes are smaller than this, we are more careful interpreting the results. We report 95% credible intervals for all parameters so that readers can evaluate potential effects of sample sizes on confidence in the results. |

| | |
|---|---|
| Data collection | Data for the present study were compiled from secondary sources by the first author (S.E.S). Data were initially stored in Microsoft Excel spreadsheets before being converted to plain-text (CSV) files and imported to R for data processing and statistical analysis. |
| Timing and spatial scale | Data collection began in August 2019, with updates made periodically until just before the point of submission. |
| Data exclusions | Our analyses included only species with complete life history data, otherwise, no further species were excluded from the analyses. |
| Reproducibility | NA: work is not experimental. |
| Randomization | NA: work is not experimental. |
| Blinding | NA: work is not experimental. |

Did the study involve field work? ☐ Yes ☒ No

# Reporting for specific materials, systems and methods

We require information from authors about some types of materials, experimental systems and methods used in many studies. Here, indicate whether each material, system or method listed is relevant to your study. If you are not sure if a list item applies to your research, read the appropriate section before selecting a response.

## Materials & experimental systems

| n/a | Involved in the study |
|---|---|
| ☒ | ☐ Antibodies |
| ☒ | ☐ Eukaryotic cell lines |
| ☒ | ☐ Palaeontology and archaeology |
| ☒ | ☐ Animals and other organisms |
| ☒ | ☐ Clinical data |
| ☒ | ☐ Dual use research of concern |

## Methods

| n/a | Involved in the study |
|---|---|
| ☒ | ☐ ChIP-seq |
| ☒ | ☐ Flow cytometry |
| ☒ | ☐ MRI-based neuroimaging |
